# Anti-flavi: A Web Platform to Predict Inhibitors of *Flaviviruses* Using QSAR and Peptidomimetic Approaches

**DOI:** 10.3389/fmicb.2018.03121

**Published:** 2018-12-18

**Authors:** Akanksha Rajput, Manoj Kumar

**Affiliations:** Virology Discovery Unit and Bioinformatics Centre, Institute of Microbial Technology, Council of Scientific and Industrial Research (CSIR), Chandigarh, India

**Keywords:** flaviviruses, inhibitor, prediction algorithm, QSAR, peptidomimetics, machine learning techniques, support vector machine, random forest

## Abstract

*Flaviviruses* are arboviruses, which comprises more than 70 viruses, covering broad geographic ranges, and responsible for significant mortality and morbidity globally. Due to the lack of efficient inhibitors targeting flaviviruses, the designing of novel and efficient anti-flavi agents is an important problem. Therefore, in the current study, we have developed a dedicated prediction algorithm *anti-flavi*, to identify inhibition ability of chemicals and peptides against flaviviruses through quantitative structure–activity relationship based method. We extracted the non-redundant 2168 chemicals and 117 peptides from ChEMBL and AVPpred databases, respectively, with reported IC_50_ values. The regression based model developed on training/testing datasets of 1952 chemicals and 105 peptides displayed the Pearson’s correlation coefficient (PCC) of 0.87, 0.84, and 0.87, 0.83 using support vector machine and random forest techniques correspondingly. We also explored the peptidomimetics approach, in which the most contributing descriptors of peptides were used to identify chemicals having anti-flavi potential. Conversely, the selected descriptors of chemicals performed well to predict anti-flavi peptides. Moreover, the developed model proved to be highly robust while checked through various approaches like independent validation and decoy datasets. We hope that our web server would prove a useful tool to predict and design the efficient anti-flavi agents. The anti-flavi webserver is freely available at URL http://bioinfo.imtech.res.in/manojk/antiflavi.

## Introduction

According to World Health Organization, *flaviviruses* are responsible for serious outbreaks world wide and hence considered as global health burden^1^ (Liang et al., 2015; Wilder-Smith and Byass, 2016). For example, the epidemics by dengue virus, DENV (100 countries in Africa, the Eastern Mediterranean, the Americas, the Western Pacific, and South-East Asia), Zika virus, ZIKV (in 42 countries), Yellow fever virus, YFV (Angolan capital city, China), and many more are reported recently. They comprise arboviruses, which are known for their shifting epidemiology in response to the changing societal factors, e.g., population growth and urbanization (Petersen and Marfin, 2005). Among all the mosquito species, the *Aedes* mosquito species are known to have prominent role in *flaviviruses* transmission, due to their ability to thrive in diverse ecological niche (beyond their resident tropical forest niche).

The genome of *flaviviruses* comprises positive-sense, non-segmented single-stranded RNA, which ranges from 9.0 to 13 kb (Simmonds et al., 2017). It code for single long open reading frame (ORF), being flanked by 5′ end (methylated nucleotide cap) and 3′ end (non-polyadenylated) and forms secondary structures for genome replication (Bollati et al., 2010). Further, the ORF codes for single large polyprotein, which is processed by host proteases and resulted in 10 proteins including structural (3) and non-structural (7). The three structural proteins are capsid (C), premembrane/membrame (prM/M), and envelop (E), whereas the seven non-structural proteins are NS1, NS2A, NS2B, NS3, NS4A, NS4B, and NS5 (Blitvich and Firth, 2017).

Various inhibitors like chemicals, peptides, and peptidomimetics have been designed against the *flaviviruses* to target their stages and proteins. For example, NITD-448 (Lim et al., 2013) inhibits E protein-mediated membrane fusion, P02 hampers the viral replication (Zhou et al., 2008), in DENV. The DN59 inhibits the flaviviral infection by interacting with viral particles (Lok et al., 2012); ST-148 is an active compound against all four DENV serotypes (Byrd et al., 2013); BP13944 (Yang et al., 2014), keto amides (Steuer et al., 2011), is known as dengue protease inhibitor; ivermectin targets the helicase activity of DENV, YFV, and JEV (Mastrangelo et al., 2012; Lai et al., 2017). Further, the NITD-618 is an effective NS4B inhibitor against all DENV serotypes (Lim et al., 2013); ribavirin impedes the DENV methyltransferase and HCV replication (Chang et al., 2011; Tomlinson and Watowich, 2011). Moreover, the NITD 008 and NITD 203 are RNA-dependent RNA polymerase inhibitors and target all the four serotype of DENV, WNV, and YFV (Caillet-Saguy et al., 2014). Lycorine displays the antiviral activity against many *flaviviruses* like YFV, WNV, and DENV-1 (Harms et al., 1991; Caillet-Saguy et al., 2014). Despite several inhibitors tested, only a few are proved efficient against the circulating mutant strains of viruses.

In literature, limited computational resources are available for predicting antiviral potential of any compound. Our group has been developing various web servers *viz.* AVPpred for predicting the effective antiviral peptides (Thakur et al., 2012), AVP-IC50Pred dedicated to identify the antiviral activity of a peptide based on the half life inhibitory concentration (Qureshi et al., 2015). Likewise, AVCpred platform was designed to predict general antiviral compounds (Qureshi et al., 2017) and HIVProtI for predicting and designing inhibitors specifically against Human Immunodeficiency Virus proteins (Qureshi et al., 2018). Since, *flaviviruses* have been emerged as worldwide threat, affecting more than 50% population globally (∼40% infected by DENV alone) (Holbrook, 2017). Therefore, there is a need to accelerate the development of efficient therapeutics. Hence, in current study we are providing *anti-flavi*, a web platform for prediction and designing of novel antiviral compounds specifically against *flaviviruses*.

## Materials and Methods

### Data Collection

For the development of predictive models, the flaviviral inhibitors were extracted from ChEMBL database (Gaulton et al., 2017), whereas the anti-flaviviral peptides were retrieved from AVPdb database (Qureshi et al., 2014). The ChEMBL is a comprehensive repository, which contains manually curated bioactive molecules possessing drug-like properties. It has been previously utilized for development of various algorithms, e.g., AVCpred (Qureshi et al., 2017), HIVProtI (Qureshi et al., 2018), CLC-Pred (Lagunin et al., 2018), and Pred-hERG (Braga et al., 2015). The AVPdb database is a comprehensive database of experimentally verified antiviral peptides (AVPs) and was earlier utilized for various algorithm like AVP-IC_50_Pred (Qureshi et al., 2015).

In the current study, we fetched the data for the inhibitors (chemicals and peptides) designed to “target” whole “organism.” The chemicals against whole organism were extracted by using specific keywords like “Dengue virus,” “Hepatitis C virus,” “West Nile Virus,” “Yellow Fever virus,” and “Japanese encephalitis virus.” Majority of the inhibition profile was reported in the form of half maximal inhibitory concentration, i.e., IC_50_, therefore we preceded our study with it. Likewise the anti-flaviviral peptides were extracted from AVPdb database with inhibition profile as the half maximal inhibitory concentration.

Initially, we obtained 65, 2038, 33, 22, 10 inhibitors against DENV (serotype 1–4), HCV, WNV, YFV, and JEV whereas 117 unique anti-flaviviral peptides. Finally, after filtering the anti-flaviviral inhibitors with relevant information and removing the redundant entries we acquired 2168 chemicals and 117 peptides (length 7 to 25 amino acids), respectively. The regression-based models were constructed on the negative logarithm of half maximal inhibitory concentration (pIC_50_ = -log_10_ (IC_50_(M))) (Kalliokoski et al., 2013; Zhou et al., 2015; Bag and Ghorai, 2016). For the development of prediction algorithm, the complete dataset was sub-divided (in triplicate) into training/testing (90%) and independent validation (10%) sets. Later, out of the three, one of the dataset set was used for algorithm development.

### Quantitative Structure Activity Relationship Based Model Development

The quantitative structure–activity relationship (QSAR) is a mathematical relationship between a biological activity and physiochemical property of any compound (Cherkasov et al., 2014). It uses various descriptors that represent the chemical characteristics of a molecule in numerical form, i.e., 1D, 2D, and 3D. We utilized the PaDEL software to extract out various molecular descriptors and fingerprints (Yap, 2011). Further, the descriptors were used for model development of anti-flaviviral compounds. Initially, the PaDEL resulted in 16384 descriptors included in 2D, 3D, and fingerprints categories. This strategy was further employed for the algorithm development in various previous studies (Qureshi et al., 2017, 2018; Rajput et al., 2018).

### Format Conversion

We performed format conversion before extracting the PaDel descriptors, in order to get the 3D descriptors along with 2D and fingerprints. In case of chemicals, the retrieved SMILES from ChEMBL were translated to SDF format through obabel software (O’Boyle et al., 2011). Whereas the anti-flavi peptides were in the form of amino acid sequences, which were firstly converted to pdb using pepstrmod (Singh et al., 2015) software with length 7 to 25 amino acids. Later on the pdbs were converted to SDF format using obabel software. We proceeded for the pdb to sdf conversion because the pdb format does not providing the complete descriptors as compared to sdf of the peptides.

### Ten-Fold Cross Validation

Initially, the model was developed on the training/testing by sub grouping into 10 almost equal parts. Of the 10 subgroups, single part is retained for testing while remaining nine was utilized for training purpose. This process was iterated 10 times, and every subgroup got the chance to be testing dataset. Further, for checking the performance of developed model the accuracy of all the 10 iterations were averaged out (Rajput et al., 2015; Thakur et al., 2016). Finally, the developed model on training/testing data set was cross-evaluated independent validation dataset.

### Support Vector Machine

For developing the regression-based predictive models, we used support vector machine (SVM) learning algorithm (Hearst, 1998). In regression mode, the SVM works on defining the function (the loss function/epsilon intensive), which ignores errors and situated within the specific distance boundaries of the actual value (Bouboulis et al., 2015). The support vector regression (SVR) is of two types, i.e., linear and non-linear. However, the non-linear SVR is much more complex as it employed kernel approach to address curse of the dimensionality. We employed SVM*^light^* module of support vector machine to develop all the models.

### Random Forest

Random forest (RF) is an ensemble-learning method that works on the basis of decision tree model with bootstrapping algorithm. First, the decision tree was made from training data sets and the classes of unknown sample is assigned either according to the mode of classes in classification or mean prediction for regression based data sets. RF was used through Waikato Environment for Knowledge Analysis (WEKA) package in prediction model development (Frank et al., 2004).

### Feature Selection

Feature selection is an important technique to extract out the best contributing features from the existing features. We implemented WEKA package for feature selection, initially the RemoveUseless filter were used for preprocessing. Further, the attributes were selected through CFsSubsetEval (*attribute evaluator*) and BestFirst (*search method*) (Frank et al., 2004). Finally, we got best representative features (relevant) for all the models.

### Performance Measure

The performance of the QSAR developed models was evaluated using correlation coefficient (R, PCC).

*Pearson’s correlation coefficient* (*R*) or bivariate correlation determine the association between two variables (actual and predicted) and calculated by the formula:

$$R=\frac{n\sum_{n\;=\;1}^{n}E_{i}^{act}E_{i}^{pred}-\sum_{n\;=\;1}^{n}E_{i}^{act}\sum_{n\;=\;1}^{n}E_{i}^{pred}}{\sqrt{n\sum_{n\;=\;1}^{n}{\left(E_{i}^{act}\right)}^{2}-{\left(\sum_{n\;=\;1}^{n}E_{i}^{act}\right)}^{2}}-\begin{array}{r} \overset{}{\sqrt{n\sum_{n\;=\;1}^{n}{\left(E_{i}^{pred}\right)}^{2}-{\left(\sum_{n\;=\;1}^{n}E_{i}^{pred}\right)}^{2}}} \end{array}}$$

Its value ranges from +1 to -1, +1 means the two variables are positively correlated whereas -1 depicts the negative correlation between two variables, here, *n*, 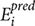, and 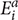
ct are size of the data set, predicted, and actual efficiencies.

### Model Performance

We checked the appropriateness of the developed models by plotting the actual v/s predicted inhibition (Qureshi et al., 2018). The plot was constructed on the actual and predicted values of training/testing as well as independent validation data sets. The scatter plot was used to depict the relationship between both the values. The best predictive ability of model is depicted by the localization of the points of actual and predicted values on/nearest to the trend line.

### Decoy Set

We used decoy set to check the robustness of our developed models. There were few tools like DUD (Huang et al., 2006), DecoyFinder (Cereto-Massague et al., 2012), and RADER (Wang et al., 2017) for designing the decoys of the chemicals. In our study, the decoys were generated from the latest tool, i.e., RApid DEcoy Retriever (RADER) software (Wang et al., 2017) against the 2168 anti-flavi chemicals with similar 1D physicochemical properties but different 2D topology.

### Clustering

We performed clustering using ChemMine tool (Backman et al., 2011). We used multidimensional scaling clustering method by both 2D and 3D method with cutoff similarity of 0.4. However, the clustering of the peptide sequences was dome using CLuster ANalysis of Sequences (CLANS) software (Frickey and Lupas, 2004), which performs *all-against-all* BLAST search.

## Results

### Feature Selection

The 16,384 features of anti-flavi chemicals and peptides were subjected to feature selection, which resulted in 8700 and 3822 features for chemicals and peptides, respectively, after the preprocessing by RemoveUseless filter. Further, the 8700 and 3822 features were processed using CfssubsetEval and BestFirst attribute selector and reduced to 124 and 19 features against chemicals and peptides correspondingly. The detailed information of all the selected descriptors of chemicals and peptides are provided in **Supplementary Tables S1**, **S2**, respectively. The models were developed using these reduced and relevant features.

### Performance of QSAR-Based Models

The 2168 anti-flavi chemicals were divided into training/testing and independent validation data sets with 1952 and 216 sequences, respectively, through randomization method. The best performing model displayed the correlation of 0.87 and 0.87 through SVM and RF machine learning technique during 10-fold cross-validation (**Table 1**). Whereas, the independent validation data set showed the correlation of 0.87 and 0.86 correspondingly with developed model during the cross-validation through SVM and RF techniques (detailed in **Supplementary Table S3**).

**Table 1:** Performance of training/testing and independent validation data sets of anti-flavi chemicals and peptides on 10-fold cross validation using Support Vector Machine and Random Forest techniques.

				Training/testing		Independent validation	
Data	Descriptors	Features	MLTs	PCC	Data set	PCC	Data set
Chemicals	16383	124	SVM	0.87	TESTSET = 1952	0.87	TESTSET = 216
Peptides	16383	19	SVM	0.84	TESTSET = 105	0.84	TESTSET = 12
Chemicals	16383	124	RF	0.87	TESTSET = 1952	0.86	TESTSET = 216
Peptides	16383	19	RF	0.83	TESTSET = 105	0.86	TESTSET = 12

*SVM, support vector machine; RF, random forest; PCC, Pearson’s correlation coefficient*.

The 117 anti-flavi peptides were grouped into 112 sequences as training/testing and 15 as independent validation data sets. Out of the three randomized models, the best one achieved correlation of 0.84 and 0.83, respectively, using SVM and RF techniques during 10-fold cross-validation on training/testing data sets (**Table 1**). While, the independent validation data set displayed the correlation of 0.84 and 0.86 correspondingly on RF and SVM techniques (detailed in **Supplementary Table S4**).

### Model Performance

**FIGURE 1:**
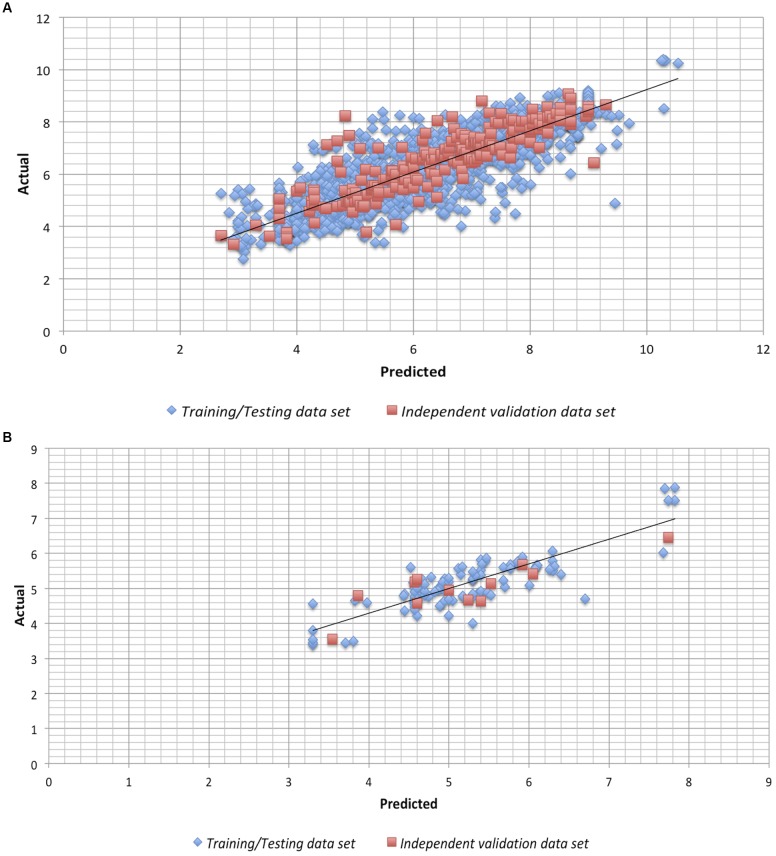
Scatter plot for Actual vs. Predicted inhibition for the independent validation data set on the Support vector machine developed models on **(A)** anti-flavi chemicals and **(B)** anti-flavi peptides.

**FIGURE 2:**
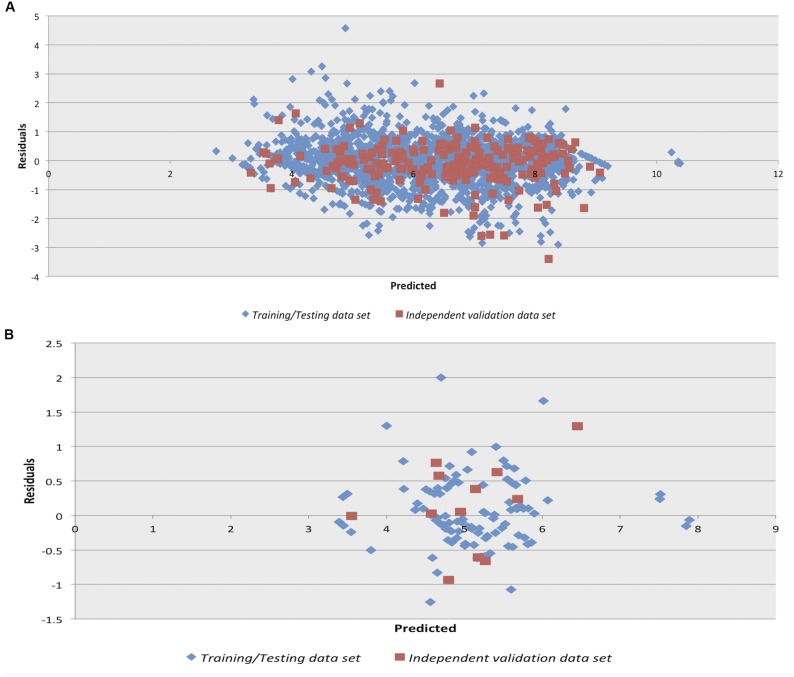
Residual plot for Residuals vs. Predicted inhibition for the independent validation data set on the Support vector machine developed models on **(A)** anti-flavi chemicals and **(B)** anti-flavi peptides.

We checked the robustness of the model by plotting actual v/s predicted value and residual plot of residuals v/s predicted values on independent validation data set of both chemical and peptides. The experimental v/s predicted values of independent validation dataset are shown in **Figure 1**. The plot between actual and predicted inhibition displayed the statistical significance among the pIC50 of the model on independent data sets. Maximum points found to be lie close to the origin, which shows that the model developed using training/testing data sets are robust. The scatter plot for actual v/s predicted of independent validation data set using SVM technique is provided in **Figure 1**. However, the scatter plot for actual v/s predicted inhibition efficiency of independent validation data set using RF technique is available in **Supplementary Figure S1**. Further, the residual plot also prove the robustness of the developed model as maximum points exist close to the origin line as shown in SVM (**Figure 2**) and RF (**Supplementary Figure S2**) models.

Further, the robustness of the model was checked using decoy data set. We opted top most hit of each 2168 chemicals, which resulted in 1417 decoys. The predicted pIC_50_ of the decoy is ranges from 3.03 to 7.83, as shown in **Supplementary Table S5**.

### Peptidomimetics Approach

We checked the peptidomimetics approach in the anti-flaviviral inhibitors by swapping the most contributing features of and peptides (19) and chemicals (124) among each other along with the hybrid features (143) using 10-fold cross validation through SVM technique. On employing the 124 features of chemicals on 117 anti-flavi peptides and 19 features on 2168 chemicals, we achieved the PCC of 0.53 and 0.74, respectively. Interestingly, on combining the top contributing features of anti-flavi chemicals and peptides, i.e., 143, we got the PCC of 0.83 and 0.87 on chemicals and peptides correspondingly. Detailed results are tabulate in **Table 2**.

**Table 2:** Table depicting the performance of swapped most-contributing features of chemicals and peptides over each other during 10-fold cross validation employing support vector machine.

Data	Descriptors	Features	PCC	Dataset
Chemicals	16383	19	0.74	TESTSET = 2168
Peptides	16383	124	0.53	TESTSET = 117
Chemicals	16383	143	0.87	TESTSET = 2168
Peptides	16383	143	0.83	TESTSET = 117

*PCC, Pearson’s correlation coefficient; cof-R2, coefficient of determination; RMSE, Root Means Squared Error; MAE, Mean Absolute Error*.

### Clustering

**FIGURE 3:**
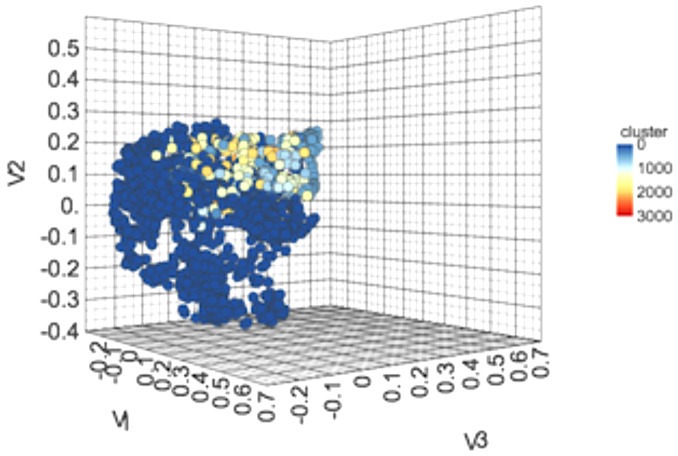
3-Dimensional plot showing the chemical spacing of 2168 anti-flavi chemicals embedded in 3D space with 58 different clusters.

We performed clustering of the anti-flavi chemicals and peptides. The clustering displayed that anti-flavi compounds are highly diverse with clustered in 58 different clusters as shown in **Figure 3**, with cutoff similarity threshold of 0.4. Additionally, the 2D plot of clusters in provided in **Supplementary Figure S3**.

**FIGURE 4:**
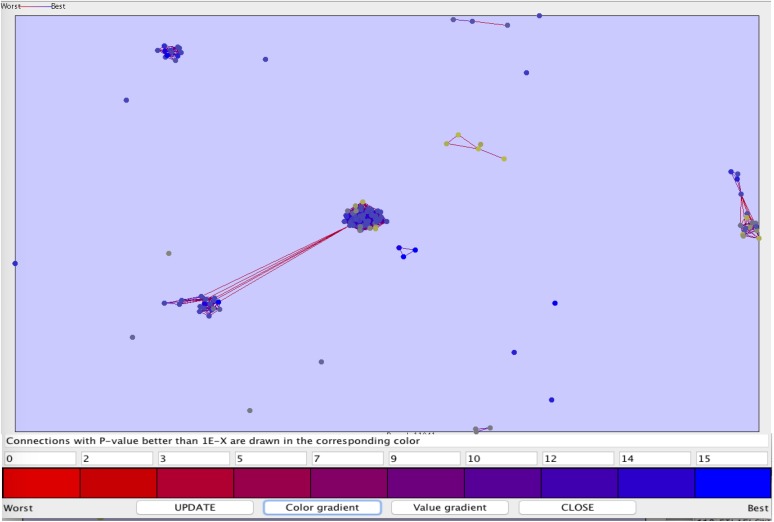
The clustering plot of the 117 anti-flavi peptides showing the clustering pattern at *p-value* 1e-90 and 0.1.

We also perform clustering of the peptide sequences, to check the diversity in out anti-flavi data sets (as shown in **Figure 4**). The *p*-value range for the clustering was set between 1e-90 and 0.1, most of the peptides were singleton. At such a stringent *p*-value we get only 10 clusters, rest sequences were found in unclustered.

### Webserver

*Anti-flavi* integrates SVM and RF predictive models to identify the inhibition efficiency of any chemical or peptides using QSAR-based approaches. For the prediction of anti-flavi chemicals, the user can provide input in form of multiple sdf formats and the output would be available in tabulated form with information of SMILES, 2D structure, important chemical descriptors, and inhibition efficiency. Whereas, for predicting the flaviviral inhibition potential of peptides the input would be provided in form of pdb format, which further led to the output as percentage inhibition of the peptide and other specifications like SMILES, 2-D image, and descriptors. As the calculation of unknown chemicals and peptides usually took 2–5 min, so the user can note the job id and retrieve the results any time using “check job status” page.

**FIGURE 5:**
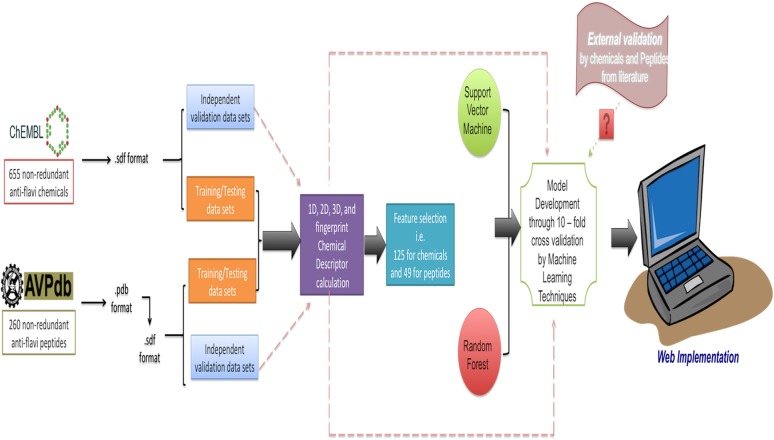
Architecture of anti-flavi web server.

The anti-flavi webservers also displayed the clustering analyses of both chemicals and peptides under the “analysis” portion. Moreover, we are also providing the format conversion facility, where the user can draw/paste the structure and get the output in form of SMILES, sdf, and mol format. The overall architecture of the anti-flavi is provided in **Figure 5**.

## Discussion

*Flaviviruses* emerged as an expanding threat to human health globally (Daep et al., 2014). Various efforts has been made to develop an effective anti-flavi drugs aiming specific replication, structural, non-structural, and host protein, as well as non-specific targets, etc (Sampath and Padmanabhan, 2009; Bollati et al., 2010; García et al., 2017). To tackle the severity of the RNA viruses, the European Union released VIZIER (Coutard and Canard, 2010) and SILVER^2^ projects for drug discovery against viruses. However, various computational efforts would be useful along with the experimental ones to speed up the antiviral drug discovery process. In this regards, the present study is focused to develop first dedicated computational platform against the flavivirus.

We used anti-flavi chemicals and peptides for developing the predictive models. Further, the peptidomimetics approach was also explored in addition to the individual chemicals and peptides. Interestingly, the performance of the developed models on chemicals is more than peptide and peptidomimetics. Additionally robustness of the developed models was also cross checked by plotting actual v/s predicted inhibition values of training/testing and independent validation data set. Finally, the predictive models proved to be statistically significant, which depicts their ability to predict any unknown agent as anti-flaviviral with high efficiency (Fatemi et al., 2015).

The concept of peptidomimetics is evidenced to be successful among the drug inhibitors, few examples of peptidomimetics were also reported against WNV (Lim et al., 2011; Hammamy et al., 2013), HIV (Kazmierski et al., 2006), etc. Our study also demonstrated the same, as we achieved good performance though most contributing features of chemicals on the peptides and vice versa. Intriguingly, the performance of the models increases when the most contributing features of chemicals and peptides were used together. Therefore, our study suggests that the concept of peptidomimetics can also be implemented in the anti-flaviviral agents.

We evaluated the performance of the models through independent validation and decoy data set. As, the developed predictive models also showed good performance on both independent validation and decoy data set, which further proves their robustness. We also tried to compare our algorithm with existing one, but didn’t able to perform direct comparison, due to lack of any method for anti-flaviviral agents.

The diversification of the chemicals and peptides were also explored using different clustering methods for both type of agents. The clustering analyses displayed high level of diversification among the anti-flavi agents at statistically significant conditions. Majority of chemicals and peptides tend to remain un-clustered rather than showing similarity through cluster forming tendency.

The effective inhibitors against *flaviviruses* are the need of the hour. The incorporation of computational approach with experimental one would definitely speed up the process of anti-flavi agents’ discovery. We used 10-fold cross validation to develop a robust prediction algorithm, which was further cross validated with independent validation as well as decoy data set. We, first time, incorporate peptidomimetics approach in prediction algorithm against *flaviviruses*. Therefore, this computational method would be highly beneficial to microbiologists and virologists, working hard to develop a novel and effective antiviral agents. This algorithm can be used to filter out the highly effective anti-flavi agents, which can be tested directly in experimental lab, rather than doing initial high through put screening. The limitation of our study is that the predictive models were developed on major flaviviral species rather than all, e.g., HCV, DENV, ZIKV, and WNV.

## Author Contributions

MK conceived the idea and helped in overall supervision. AR and MK performed the data collection, model development, analyses, and wrote the manuscript. AR executed the web server.

## Conflict of Interest Statement

The authors declare that the research was conducted in the absence of any commercial or financial relationships that could be construed as a potential conflict of interest.

## Footnotes

1 http://www.who.int/csr/disease/epidemic-focus/flavivirus-epidemics/en/

2 https://www.silverpcp.eu/project-overview/

## References

[B1] Backman T. W. H. Cao Y. Girke T. (2011). ChemMine tools: an online service for analyzing and clustering small molecules. *Nucleic Acids Res.* 39 W486–W491. 10.1093/nar/gkr320 21576229 PMC3125754

[B2] Bag A. Ghorai P. K. (2016). Development of quantum chemical method to calculate half maximal inhibitory concentration (IC50). *Mol. Inform.* 35 199–206. 10.1002/minf.201501004 27492086

[B3] Blitvich B. J. Firth A. E. (2017). A review of flaviviruses that have no known arthropod vector. *Viruses* 9:E154. 10.3390/v9060154 28635667 PMC5490829

[B4] Bollati M. Alvarez K. Assenberg R. Baronti C. Canard B. Cook S. (2010). Structure and functionality in flavivirus NS-proteins: perspectives for drug design. *Antiviral Res.* 87 125–148. 10.1016/j.antiviral.2009.11.009 19945487 PMC3918146

[B5] Bouboulis P. Theodoridis S. Mavroforakis C. Evaggelatou-Dalla L. (2015). Complex support vector machines for regression and quaternary classification. *IEEE Trans. Neural Netw. Learn. Syst.* 26 1260–1274. 10.1109/tnnls.2014.2336679 25095266

[B6] Braga R. C. Alves V. M. Silva M. F. Muratov E. Fourches D. Liao L. M. (2015). Pred-hERG: a novel web-accessible computational tool for predicting cardiac toxicity. *Mol. Inform.* 34 698–701. 10.1002/minf.201500040 27490970 PMC5720373

[B7] Byrd C. M. Dai D. Grosenbach D. W. Berhanu A. Jones K. F. Cardwell K. B. (2013). A novel inhibitor of dengue virus replication that targets the capsid protein. *Antimicrob. Agents Chemother.* 57 15–25. 10.1128/aac.01429-12 23070172 PMC3535982

[B8] Caillet-Saguy C. Lim S. P. Shi P. Y. Lescar J. Bressanelli S. (2014). Polymerases of hepatitis C viruses and flaviviruses: structural and mechanistic insights and drug development. *Antiviral Res.* 105 8–16. 10.1016/j.antiviral.2014.02.006 24561230

[B9] Cereto-Massague A. Guasch L. Valls C. Mulero M. Pujadas G. Garcia-Vallve S. (2012). DecoyFinder: an easy-to-use python GUI application for building target-specific decoy sets. *Bioinformatics* 28 1661–1662. 10.1093/bioinformatics/bts249 22539671

[B10] Chang J. Schul W. Butters T. D. Yip A. Liu B. Goh A. (2011). Combination of alpha-glucosidase inhibitor and ribavirin for the treatment of dengue virus infection in vitro and in vivo. *Antiviral Res.* 89 26–34. 10.1016/j.antiviral.2010.11.002 21073903 PMC3018560

[B11] Cherkasov A. Muratov E. N. Fourches D. Varnek A. Baskin I. I. Cronin M. (2014). QSAR modeling: where have you been? Where are you going to? *J. Med. Chem.* 57 4977–5010. 10.1021/jm4004285 24351051 PMC4074254

[B12] Coutard B. Canard B. (2010). The VIZIER project: overview; expectations; and achievements. *Antiviral Res.* 87 85–94. 10.1016/j.antiviral.2010.02.326 20226212 PMC7114346

[B13] Daep C. A. Muñoz-Jordán J. L. Eugenin E. A. (2014). Flaviviruses, an expanding threat in public health: focus on Dengue, West Nile, and Japanese encephalitis virus. *J. Neurovirol.* 20 539–560. 10.1007/s13365-014-0285-z 25287260 PMC4331079

[B14] Fatemi M. H. Heidari A. Gharaghani S. (2015). QSAR prediction of HIV-1 protease inhibitory activities using docking derived molecular descriptors. *J. Theor. Biol.* 369 13–22. 10.1016/j.jtbi.2015.01.008 25600056

[B15] Frank E. Hall M. Trigg L. Holmes G. Witten I. H. (2004). Data mining in bioinformatics using Weka. *Bioinformatics* 20 2479–2481. 10.1093/bioinformatics/bth261 15073010

[B16] Frickey T. Lupas A. (2004). CLANS: a Java application for visualizing protein families based on pairwise similarity. *Bioinformatics* 20 3702–3704. 10.1093/bioinformatics/bth444 15284097

[B17] García L. L. Padilla L. Castaño J. C. (2017). Inhibitors compounds of the flavivirus replication process. *Virol. J.* 14:95. 10.1186/s12985-017-0761-1 28506240 PMC5433246

[B18] Gaulton A. Hersey A. Nowotka M. Bento A. P. Chambers J. Mendez D. (2017). The ChEMBL database in 2017. *Nucleic Acids Res.* 45 D945–D954. 10.1093/nar/gkw1074 27899562 PMC5210557

[B19] Hammamy M. Z. Haase C. Hammami M. Hilgenfeld R. Steinmetzer T. (2013). Development and characterization of new peptidomimetic inhibitors of the West Nile virus NS2B-NS3 protease. *ChemMedChem* 8 231–241. 10.1002/cmdc.201200497 23307694

[B20] Harms G. Pedrosa C. Omena S. Feldmeier H. Zwingenberger K. (1991). Natural killer cell activity in visceral leishmaniasis. *Trans. R. Soc. Trop. Med. Hyg.* 85 54–55. 10.1016/0035-9203(91)90154-Q 1906206

[B21] Hearst M. A. (1998). Support vector machines. *IEEE Intell. Syst.* 13 18–28. 10.1109/5254.708428

[B22] Holbrook M. R. (2017). Historical perspectives on flavivirus research. *Viruses* 9:E97. 10.3390/v9050097 28468299 PMC5454410

[B23] Huang N. Shoichet B. K. Irwin J. J. (2006). Benchmarking sets for molecular docking. *J. Med. Chem.* 49 6789–6801. 10.1021/jm0608356 17154509 PMC3383317

[B24] Kalliokoski T. Kramer C. Vulpetti A. Gedeck P. (2013). Comparability of mixed IC(5)(0) data - a statistical analysis. *PLoS One* 8:e61007. 10.1371/journal.pone.0061007 23613770 PMC3628986

[B25] Kazmierski W. M. Kenakin T. P. Gudmundsson K. S. (2006). Peptide, peptidomimetic and small-molecule drug discovery targeting HIV-1 host-cell attachment and entry through gp120, gp41, CCR5 and CXCR4. *Chem. Biol. Drug Des.* 67 13–26. 10.1111/j.1747-0285.2005.00319.x 16492145

[B26] Lagunin A. A. Dubovskaja V. I. Rudik A. V. Pogodin P. V. Druzhilovskiy D. S. Gloriozova T. A. (2018). CLC-Pred: a freely available web-service for in silico prediction of human cell line cytotoxicity for drug-like compounds. *PLoS One* 13:e0191838. 10.1371/journal.pone.0191838 29370280 PMC5784992

[B27] Lai J. H. Lin Y. L. Hsieh S. L. (2017). Pharmacological intervention for dengue virus infection. *Biochem. Pharmacol.* 129 14–25. 10.1016/j.bcp.2017.01.005 28104437

[B28] Liang G. Gao X. Gould E. A. (2015). Factors responsible for the emergence of arboviruses; strategies, challenges and limitations for their control. *Emerg. Microbes Infect.* 4:e18. 10.1038/emi.2015.18 26038768 PMC4395659

[B29] Lim H. A. Joy J. Hill J. San Brian Chia C. (2011). Novel agmatine and agmatine-like peptidomimetic inhibitors of the West Nile virus NS2B/NS3 serine protease. *Eur. J. Med. Chem.* 46 3130–3134. 10.1016/j.ejmech.2011.04.055 21565434

[B30] Lim S. P. Wang Q. Y. Noble C. G. Chen Y. L. Dong H. Zou B. (2013). Ten years of dengue drug discovery: progress and prospects. *Antiviral Res.* 100 500–519. 10.1016/j.antiviral.2013.09.013 24076358

[B31] Lok S. M. Costin J. M. Hrobowski Y. M. Hoffmann A. R. Rowe D. K. Kukkaro P. (2012). Release of dengue virus genome induced by a peptide inhibitor. *PLoS One* 7:e50995. 10.1371/journal.pone.0050995 23226444 PMC3511436

[B32] Mastrangelo E. Pezzullo M. De Burghgraeve T. Kaptein S. Pastorino B. Dallmeier K. (2012). Ivermectin is a potent inhibitor of flavivirus replication specifically targeting NS3 helicase activity: new prospects for an old drug. *J. Antimicrob. Chemother.* 67 1884–1894. 10.1093/jac/dks147 22535622 PMC3888155

[B33] O’Boyle N. M. Banck M. James C. A. Morley C. Vandermeersch T. Hutchison G. R. (2011). Open babel: an open chemical toolbox. *J. Cheminform.* 3:33. 10.1186/1758-2946-3-33 21982300 PMC3198950

[B34] Petersen L. R. Marfin A. A. (2005). Shifting epidemiology of flaviviridae. *J. Travel Med.* 12(Suppl. 1), S3–S11. 10.2310/7060.2005.12052 16225801

[B35] Qureshi A. Kaur G. Kumar M. (2017). AVCpred: an integrated web server for prediction and design of antiviral compounds. *Chem. Biol. Drug Des.* 89 74–83. 10.1111/cbdd.12834 27490990 PMC7162012

[B36] Qureshi A. Rajput A. Kaur G. Kumar M. (2018). HIVprotI: an integrated web based platform for prediction and design of HIV proteins inhibitors. *J. Cheminform.* 10:12. 10.1186/s13321-018-0266-y 29524011 PMC5845081

[B37] Qureshi A. Tandon H. Kumar M. (2015). AVP-IC50 Pred: multiple machine learning techniques-based prediction of peptide antiviral activity in terms of half maximal inhibitory concentration (IC50). *Biopolymers* 104 753–763. 10.1002/bip.22703 26213387 PMC7161829

[B38] Qureshi A. Thakur N. Tandon H. Kumar M. (2014). AVPdb: a database of experimentally validated antiviral peptides targeting medically important viruses. *Nucleic Acids Res.* 42 D1147–D1153. 10.1093/nar/gkt1191 24285301 PMC3964995

[B39] Rajput A. Gupta A. K. Kumar M. (2015). Prediction and analysis of quorum sensing peptides based on sequence features. *PLoS One* 10:e0120066. 10.1371/journal.pone.0120066 25781990 PMC4363368

[B40] Rajput A. Thakur A. Sharma S. Kumar M. (2018). aBiofilm: a resource of anti-biofilm agents and their potential implications in targeting antibiotic drug resistance. *Nucleic Acids Res.* 46 D894–D900. 10.1093/nar/gkx1157 29156005 PMC5753393

[B41] Sampath A. Padmanabhan R. (2009). Molecular targets for flavivirus drug discovery. *Antiviral Res.* 81 6–15. 10.1016/j.antiviral.2008.08.004 18796313 PMC2647018

[B42] Simmonds P. Becher P. Bukh J. Gould E. A. Meyers G. Monath T. (2017). ICTV virus taxonomy profile: flaviviridae. *J. Gen. Virol.* 98 2–3. 10.1099/jgv.0.000672 28218572 PMC5370391

[B43] Singh S. Singh H. Tuknait A. Chaudhary K. Singh B. Kumaran S. (2015). PEPstrMOD: structure prediction of peptides containing natural, non-natural and modified residues. *Biol. Direct* 10:73. 10.1186/s13062-015-0103-4 26690490 PMC4687368

[B44] Steuer C. Gege C. Fischl W. Heinonen K. H. Bartenschlager R. Klein C. D. (2011). Synthesis and biological evaluation of alpha-ketoamides as inhibitors of the Dengue virus protease with antiviral activity in cell-culture. *Bioorg. Med. Chem.* 19 4067–4074. 10.1016/j.bmc.2011.05.015 21641807

[B45] Thakur A. Rajput A. Kumar M. (2016). MSLVP: prediction of multiple subcellular localization of viral proteins using a support vector machine. *Mol. Biosyst.* 12 2572–2586. 10.1039/c6mb00241b 27272007

[B46] Thakur N. Qureshi A. Kumar M. (2012). AVPpred: collection and prediction of highly effective antiviral peptides. *Nucleic Acids Res.* 40 W199–W204. 10.1093/nar/gks450 22638580 PMC3394244

[B47] Tomlinson S. M. Watowich S. J. (2011). Anthracene-based inhibitors of dengue virus NS2B-NS3 protease. *Antiviral Res.* 89 127–135. 10.1016/j.antiviral.2010.12.006 21185332 PMC3026091

[B48] Wang L. Pang X. Li Y. Zhang Z. Tan W. (2017). RADER: a RApid DEcoy retriever to facilitate decoy based assessment of virtual screening. *Bioinformatics* 33 1235–1237. 10.1093/bioinformatics/btw783 28011765

[B49] Wilder-Smith A. Byass P. (2016). The elusive global burden of dengue. *Lancet Infect. Dis.* 16 629–631. 10.1016/s1473-3099(16)00076-1 26874620

[B50] Yang C. C. Hu H. S. Wu R. H. Wu S. H. Lee S. J. Jiaang W. T. (2014). A novel dengue virus inhibitor, BP13944, discovered by high-throughput screening with dengue virus replicon cells selects for resistance in the viral NS2B/NS3 protease. *Antimicrob. Agents Chemother.* 58 110–119. 10.1128/aac.01281-13 24145533 PMC3910792

[B51] Yap C. W. (2011). PaDEL-descriptor: an open source software to calculate molecular descriptors and fingerprints. *J. Comput. Chem.* 32 1466–1474. 10.1002/jcc.21707 21425294

[B52] Zhou N. Xu Y. Liu X. Wang Y. Peng J. Luo X. (2015). Combinatorial pharmacophore-Based 3D-QSAR analysis and virtual screening of FGFR1 inhibitors. *Int. J. Mol. Sci.* 16 13407–13426. 10.3390/ijms160613407 26110383 PMC4490501

[B53] Zhou Z. Khaliq M. Suk J. E. Patkar C. Li L. Kuhn R. J. (2008). Antiviral compounds discovered by virtual screening of small-molecule libraries against dengue virus E protein. *ACS Chem. Biol.* 3 765–775. 10.1021/cb800176t 19053243 PMC2782732

