# Supplementary material for: Anti-flavi: A Web Platform to Predict Inhibitors of Flaviviruses Using QSAR and Peptidomimetic Approaches
Source: Front Microbiol. 2018 Dec 18;9:3121. doi: 10.3389/fmicb.2018.03121 (PMC6305493; doi:10.3389/fmicb.2018.03121)
Supplement: Supplementary file 1 [file Table_1.DOCX]

**Anti-flavi: A web platform to predict inhibitors of *flaviviruses* using QSAR and peptidomimetic approaches**

Akanksha Rajput and Manoj Kumar*

Virology Discovery Unit and Bioinformatics Centre, Institute of Microbial Technology, Council of Scientific and Industrial Research (CSIR), Sector 39-A, Chandigarh-160036, India

*To whom correspondence should be addressed. Tel: +91 172 6665453; Fax: +91 172 2690585; Email: [manojk@imtech.res.in](mailto:manojk@imtech.res.in)

**Supplementary information**

**Supplementary Figures**

**Supplementary Figure S1**. Scatter plot for Actual *vs* Predicted inhibition for the independent validation data set on the Random Forest developed models on A. anti-flavi chemicals and B. anti-flavi peptides

**Supplementary Figure S2.** Residual plot for Residuals *vs* Predicted inhibition for the independent validation data set on the Random Forest developed models on A. anti-flavi chemicals and B. anti-flavi peptides

**Supplementary Figure S3.** 2-Dimensional plot showing the chemical spacing of 655 anti-flavi chemicals embedded in 2D space with 58 different clusters

**Supplementary Table S1.** Description of the top most 124 contributing features of anti-flavi chemicals

**Supplementary Table S2.** Description of the top most 19 contributing features of anti-flavi peptides

**Supplementary Table S3.** Actual and predicted pIC_50_ of 216 independent validation data set of chemicals

**Supplementary Table S4.** Actual and predicted pIC_50_ of 12 independent validation data set of peptides

**Supplementary Table S5.** The actual and predicted pIC50 of the decoy sets

**Supplementary Figures**


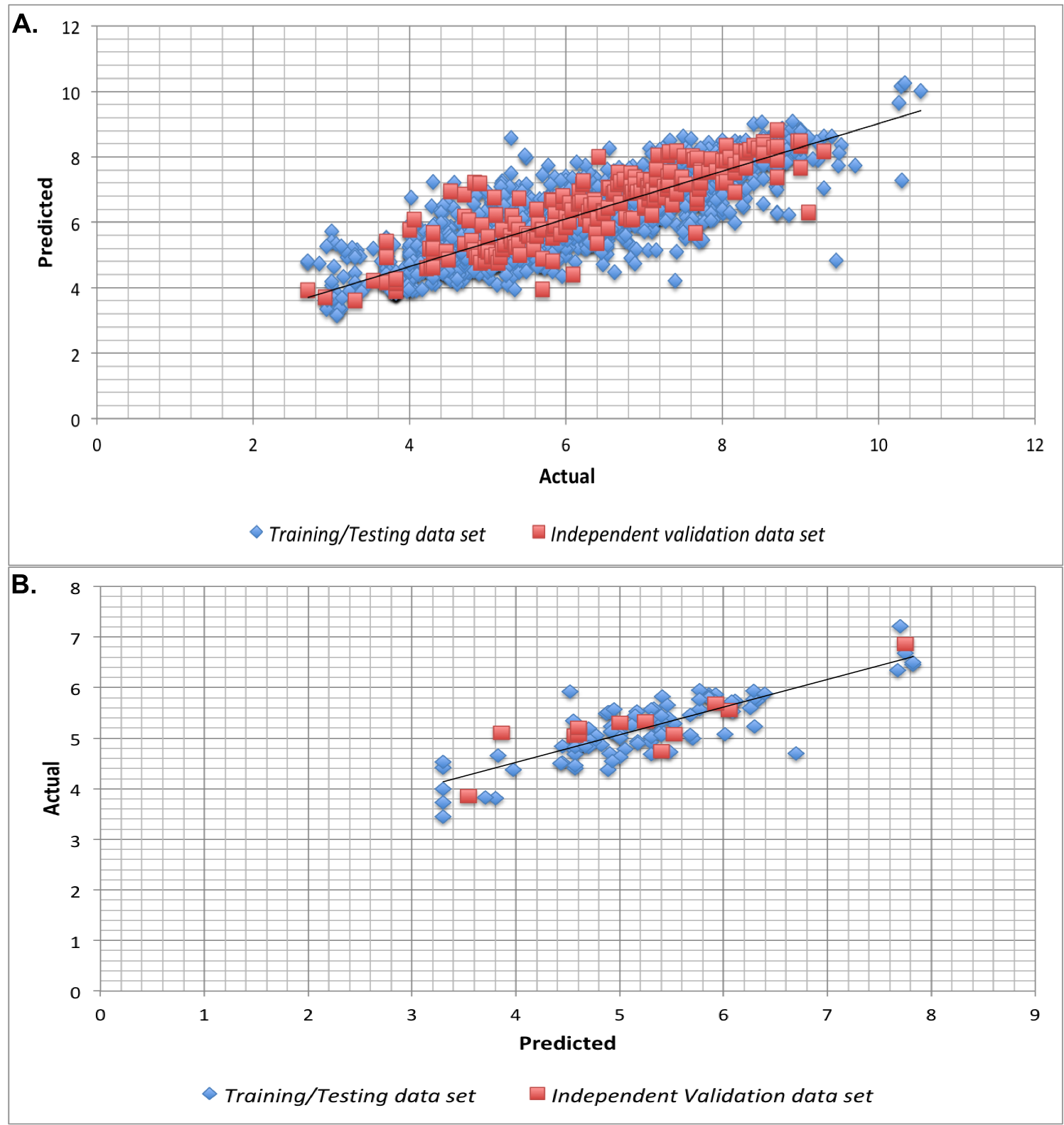


**Supplementary Figure S1**. Scatter plot for Actual *vs* Predicted inhibition for the independent validation data set on the Random Forest developed models on A. anti-flavi chemicals and B. anti-flavi peptides

**Supplementary Figure S2.** Residual plot for Residuals *vs* Predicted inhibition for the independent validation data set on the Random Forest developed models on A. anti-flavi chemicals and B. anti-flavi peptides

**
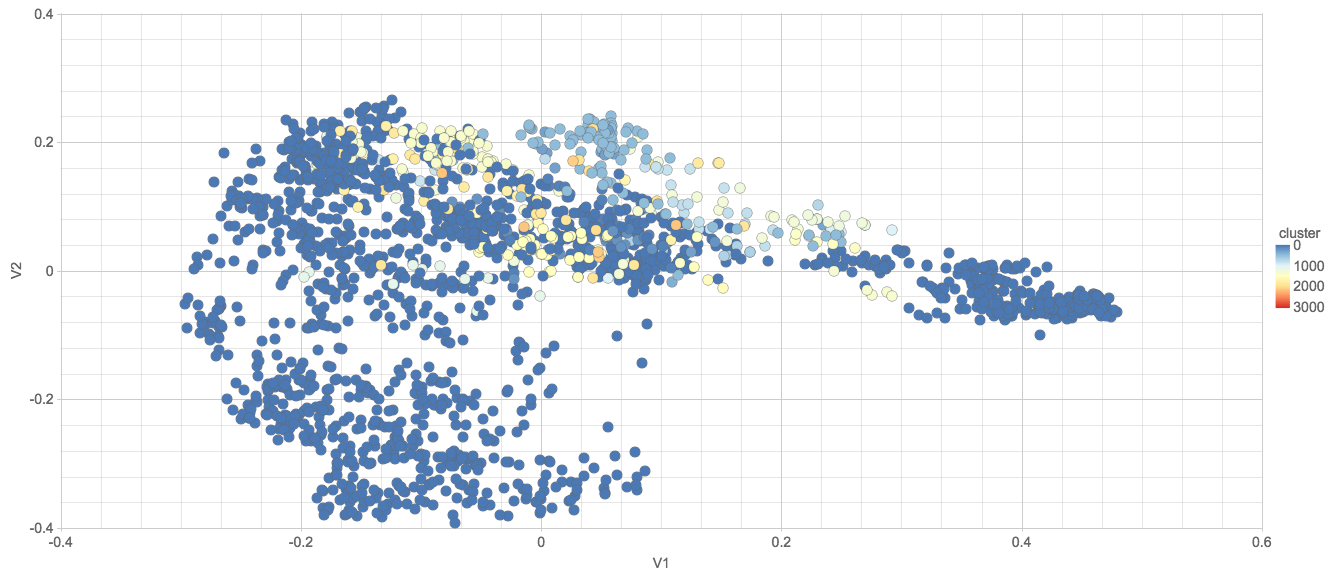
**

**Supplementary Figure S3.** 2-Dimensional plot showing the chemical spacing of 655 anti-flavi chemicals embedded in 2D space with 58 different clusters

**Supplementary Tables**

**Supplementary Table S1.** Description of the top most 124 contributing features of anti-flavi chemicals.

| Chemicals | Descripion | Type |
| --- | --- | --- |
| VE1_Dzs | Coefficient sum of the last eigenvector from Barysz matrix / weighted by I-state | 2D |
| SpMin1_Bhe | Smallest absolute eigenvalue of Burden modified matrix - n 1 / weighted by relative Sanderson electronegativities | 2D |
| SpMax2_Bhi | Largest absolute eigenvalue of Burden modified matrix - n 2 / weighted by relative first ionization potential | 2D |
| VC-4 | Valence cluster, order 4 | 2D |
| mindssC | Minimum atom-type E-State: =C< | 2D |
| IC3 | Information content index (neighborhood symmetry of 3-order) | 2D |
| FP87 | Fingerprint of length 1024 and search depth of 8 | CDK fingerprint |
| FP317 | Fingerprint of length 1024 and search depth of 8 | CDK fingerprint |
| FP466 | Fingerprint of length 1024 and search depth of 8 | CDK fingerprint |
| FP512 | Fingerprint of length 1024 and search depth of 8 | CDK fingerprint |
| FP672 | Fingerprint of length 1024 and search depth of 8 | CDK fingerprint |
| FP832 | Fingerprint of length 1024 and search depth of 8 | CDK fingerprint |
| FP915 | Fingerprint of length 1024 and search depth of 8 | CDK fingerprint |
| FP937 | Fingerprint of length 1024 and search depth of 8 | CDK fingerprint |
| ExtFP190 | Extends the Fingerprinter with additional bits describing ring features | CDK extended fingerprint |
| ExtFP216 | Extends the Fingerprinter with additional bits describing ring features | CDK extended fingerprint |
| ExtFP415 | Extends the Fingerprinter with additional bits describing ring features | CDK extended fingerprint |
| ExtFP717 | Extends the Fingerprinter with additional bits describing ring features | CDK extended fingerprint |
| ExtFP726 | Extends the Fingerprinter with additional bits describing ring features | CDK extended fingerprint |
| ExtFP803 | Extends the Fingerprinter with additional bits describing ring features | CDK extended fingerprint |
| ExtFP959 | Extends the Fingerprinter with additional bits describing ring features | CDK extended fingerprint |
| GraphFP109 | Specialized version of the Fingerprinter which does not take bond orders into account | CDK graph only fingerprint |
| GraphFP165 | Specialized version of the Fingerprinter which does not take bond orders into account | CDK graph only fingerprint |
| GraphFP255 | Specialized version of the Fingerprinter which does not take bond orders into account | CDK graph only fingerprint |
| GraphFP495 | Specialized version of the Fingerprinter which does not take bond orders into account | CDK graph only fingerprint |
| GraphFP522 | Specialized version of the Fingerprinter which does not take bond orders into account | CDK graph only fingerprint |
| GraphFP694 | Specialized version of the Fingerprinter which does not take bond orders into account | CDK graph only fingerprint |
| GraphFP702 | Specialized version of the Fingerprinter which does not take bond orders into account | CDK graph only fingerprint |
| GraphFP817 | Specialized version of the Fingerprinter which does not take bond orders into account | CDK graph only fingerprint |
| GraphFP832 | Specialized version of the Fingerprinter which does not take bond orders into account | CDK graph only fingerprint |
| GraphFP920 | Specialized version of the Fingerprinter which does not take bond orders into account | CDK graph only fingerprint |
| GraphFP942 | Specialized version of the Fingerprinter which does not take bond orders into account | CDK graph only fingerprint |
| MACCSFP163 | MACCS keys | MACCS fingerprint |
| PubchemFP408 | Pubchem fingerprint | Pubchem fingerprint |
| PubchemFP422 | Pubchem fingerprint | Pubchem fingerprint |
| PubchemFP558 | Pubchem fingerprint | Pubchem fingerprint |
| PubchemFP609 | Pubchem fingerprint | Pubchem fingerprint |
| PubchemFP637 | Pubchem fingerprint | Pubchem fingerprint |
| PubchemFP719 | Pubchem fingerprint | Pubchem fingerprint |
| SubFP8 | Presence of SMARTS Patterns for Functional Group Classification by Christian Laggner | Substructure fingerprint |
| SubFP204 | Presence of SMARTS Patterns for Functional Group Classification by Christian Laggner | Substructure fingerprint |
| KRFP13 | Presence of chemical substructures | Klekota-Roth fingerprint |
| KRFP424 | Presence of chemical substructures | Klekota-Roth fingerprint |
| KRFP578 | Presence of chemical substructures | Klekota-Roth fingerprint |
| KRFP773 | Presence of chemical substructures | Klekota-Roth fingerprint |
| KRFP837 | Presence of chemical substructures | Klekota-Roth fingerprint |
| KRFP892 | Presence of chemical substructures | Klekota-Roth fingerprint |
| KRFP992 | Presence of chemical substructures | Klekota-Roth fingerprint |
| KRFP1139 | Presence of chemical substructures | Klekota-Roth fingerprint |
| KRFP1170 | Presence of chemical substructures | Klekota-Roth fingerprint |
| KRFP1218 | Presence of chemical substructures | Klekota-Roth fingerprint |
| KRFP1404 | Presence of chemical substructures | Klekota-Roth fingerprint |
| KRFP1463 | Presence of chemical substructures | Klekota-Roth fingerprint |
| KRFP1564 | Presence of chemical substructures | Klekota-Roth fingerprint |
| KRFP1672 | Presence of chemical substructures | Klekota-Roth fingerprint |
| KRFP1826 | Presence of chemical substructures | Klekota-Roth fingerprint |
| KRFP1988 | Presence of chemical substructures | Klekota-Roth fingerprint |
| KRFP2135 | Presence of chemical substructures | Klekota-Roth fingerprint |
| KRFP2242 | Presence of chemical substructures | Klekota-Roth fingerprint |
| KRFP2386 | Presence of chemical substructures | Klekota-Roth fingerprint |
| KRFP2443 | Presence of chemical substructures | Klekota-Roth fingerprint |
| KRFP2587 | Presence of chemical substructures | Klekota-Roth fingerprint |
| KRFP2590 | Presence of chemical substructures | Klekota-Roth fingerprint |
| KRFP2651 | Presence of chemical substructures | Klekota-Roth fingerprint |
| KRFP2675 | Presence of chemical substructures | Klekota-Roth fingerprint |
| KRFP2777 | Presence of chemical substructures | Klekota-Roth fingerprint |
| KRFP2816 | Presence of chemical substructures | Klekota-Roth fingerprint |
| KRFP3014 | Presence of chemical substructures | Klekota-Roth fingerprint |
| KRFP3203 | Presence of chemical substructures | Klekota-Roth fingerprint |
| KRFP3521 | Presence of chemical substructures | Klekota-Roth fingerprint |
| KRFP3524 | Presence of chemical substructures | Klekota-Roth fingerprint |
| KRFP3568 | Presence of chemical substructures | Klekota-Roth fingerprint |
| KRFP3676 | Presence of chemical substructures | Klekota-Roth fingerprint |
| KRFP3749 | Presence of chemical substructures | Klekota-Roth fingerprint |
| KRFP4006 | Presence of chemical substructures | Klekota-Roth fingerprint |
| KRFP4008 | Presence of chemical substructures | Klekota-Roth fingerprint |
| KRFP4490 | Presence of chemical substructures | Klekota-Roth fingerprint |
| KRFP4504 | Presence of chemical substructures | Klekota-Roth fingerprint |
| KRFP4643 | Presence of chemical substructures | Klekota-Roth fingerprint |
| KRFP4666 | Presence of chemical substructures | Klekota-Roth fingerprint |
| KRFP4755 | Presence of chemical substructures | Klekota-Roth fingerprint |
| AD2D102 | Presence of atom pairs at various topological distances | 2D atom pairs |
| AD2D441 | Presence of atom pairs at various topological distances | 2D atom pairs |
| AD2D484 | Presence of atom pairs at various topological distances | 2D atom pairs |
| AD2D598 | Presence of atom pairs at various topological distances | 2D atom pairs |
| AD2D677 | Presence of atom pairs at various topological distances | 2D atom pairs |
| AD2D704 | Presence of atom pairs at various topological distances | 2D atom pairs |
| AD2D761 | Presence of atom pairs at various topological distances | 2D atom pairs |
| SubFPC96 | Count of SMARTS Patterns for Functional Group Classification by Christian Laggner | Substructure fingerprint count |
| SubFPC105 | Count of SMARTS Patterns for Functional Group Classification by Christian Laggner | Substructure fingerprint count |
| SubFPC198 | Count of SMARTS Patterns for Functional Group Classification by Christian Laggner | Substructure fingerprint count |
| SubFPC204 | Count of SMARTS Patterns for Functional Group Classification by Christian Laggner | Substructure fingerprint count |
| SubFPC292 | Count of SMARTS Patterns for Functional Group Classification by Christian Laggner | Substructure fingerprint count |
| KRFPC13 | Count of chemical substructures | Klekota-Roth fingerprint count |
| KRFPC364 | Count of chemical substructures | Klekota-Roth fingerprint count |
| KRFPC578 | Count of chemical substructures | Klekota-Roth fingerprint count |
| KRFPC773 | Count of chemical substructures | Klekota-Roth fingerprint count |
| KRFPC892 | Count of chemical substructures | Klekota-Roth fingerprint count |
| KRFPC1170 | Count of chemical substructures | Klekota-Roth fingerprint count |
| KRFPC1173 | Count of chemical substructures | Klekota-Roth fingerprint count |
| KRFPC1218 | Count of chemical substructures | Klekota-Roth fingerprint count |
| KRFPC1404 | Count of chemical substructures | Klekota-Roth fingerprint count |
| KRFPC1452 | Count of chemical substructures | Klekota-Roth fingerprint count |
| KRFPC1463 | Count of chemical substructures | Klekota-Roth fingerprint count |
| KRFPC1577 | Count of chemical substructures | Klekota-Roth fingerprint count |
| KRFPC1600 | Count of chemical substructures | Klekota-Roth fingerprint count |
| KRFPC1621 | Count of chemical substructures | Klekota-Roth fingerprint count |
| KRFPC1656 | Count of chemical substructures | Klekota-Roth fingerprint count |
| KRFPC1826 | Count of chemical substructures | Klekota-Roth fingerprint count |
| KRFPC1905 | Count of chemical substructures | Klekota-Roth fingerprint count |
| KRFPC1917 | Count of chemical substructures | Klekota-Roth fingerprint count |
| KRFPC2386 | Count of chemical substructures | Klekota-Roth fingerprint count |
| KRFPC2443 | Count of chemical substructures | Klekota-Roth fingerprint count |
| KRFPC2457 | Count of chemical substructures | Klekota-Roth fingerprint count |
| KRFPC2587 | Count of chemical substructures | Klekota-Roth fingerprint count |
| KRFPC2651 | Count of chemical substructures | Klekota-Roth fingerprint count |
| KRFPC3137 | Count of chemical substructures | Klekota-Roth fingerprint count |
| KRFPC3203 | Count of chemical substructures | Klekota-Roth fingerprint count |
| KRFPC3542 | Count of chemical substructures | Klekota-Roth fingerprint count |
| KRFPC3543 | Count of chemical substructures | Klekota-Roth fingerprint count |
| KRFPC3583 | Count of chemical substructures | Klekota-Roth fingerprint count |
| KRFPC3645 | Count of chemical substructures | Klekota-Roth fingerprint count |
| KRFPC3749 | Count of chemical substructures | Klekota-Roth fingerprint count |
| KRFPC4008 | Count of chemical substructures | Klekota-Roth fingerprint count |

**Supplementary Table S2.** Description of the top most 19 contributing features of anti-flavi peptides.

| Peptide | Descripion | Type |
| --- | --- | --- |
| GATS3e | Geary autocorrelation - lag 3 / weighted by Sanderson electronegativities | 2D |
| BCUTc-1l | nhigh lowest partial charge weighted BCUTS | 2D |
| SpMax4_Bhv | Largest absolute eigenvalue of Burden modified matrix - n 4 / weighted by relative van der Waals volumes | 2D |
| SpMax4_Bhp | Largest absolute eigenvalue of Burden modified matrix - n 4 / weighted by relative polarizabilities | 2D |
| AVP-6 | Average valence path, order 6 | 2D |
| maxHCsats | Maximum atom-type H E-State: H bonded to B, Si, P, Ge, As, Se, Sn or Pb | 2D |
| SIC4 | Structural information content index (neighborhood symmetry of 4-order) | 2D |
| BIC4 | Bond information content index (neighborhood symmetry of 4-order) | 2D |
| MDEC-11 | Molecular distance edge between all primary carbons | 2D |
| MDEN-33 | Molecular distance edge between all tertiary nitrogens | 2D |
| E1m | 1st component accessibility directional WHIM index / weighted by relative mass | 3D |
| E2v | 2nd component accessibility directional WHIM index / weighted by relative van der Waals volumes | 3D |
| FP170 | Fingerprint of length 1024 and search depth of 8 | CDK fingerprint |
| FP181 | Fingerprint of length 1024 and search depth of 8 | CDK fingerprint |
| FP424 | Fingerprint of length 1024 and search depth of 8 | CDK fingerprint |
| ExtFP904 | Extends the Fingerprinter with additional bits describing ring features | CDK extended fingerprint |
| GraphFP59 | Specialized version of the Fingerprinter which does not take bond orders into account | CDK graph only fingerprint |
| GraphFP767 | Specialized version of the Fingerprinter which does not take bond orders into account | CDK graph only fingerprint |
| PubchemFP152 | Fingerprint of length 1024 and search depth of 8 | CDK fingerprint |

**Supplementary Table S3.** Actual and predicted pIC_50_ of 216 independent validation data set of chemicals

| **CHEMBL_ID** | **SMILES** | **Actual pIC50** | **Predicted pIC50** |
| --- | --- | --- | --- |
| CHEMBL575429 | OC(=O)\C=C\c1ccc(OS(=O)(=O)O)cc1 | 2.698970004 | 3.6504459 |
| CHEMBL608973 | OC[C@H]1OC([C@H](O)[C@@H]1O)n2cnc3C(=O)N=C(NCCc4ccccc4)NC(=O)c23 | 2.919506942 | 3.3274757 |
| CHEMBL611862 | OC[C@H]1OC([C@H](O)[C@@H]1O)N2C=NC3NC(=O)NNC(=O)C23 | 3.301029996 | 4.0495852 |
| CHEMBL400748 | COC(=N)c1ncn(n1)[C@@H]2O[C@H](CO)[C@@H](O)[C@H]2O | 3.531193103 | 3.6355312 |
| CHEMBL482521 | CN(C(=O)c1cc(I)cc(I)c1O)c2ccc(Cl)c(Cl)c2 | 3.698970004 | 4.6524129 |
| CHEMBL2011045 | CC(C)c1ccc(NC(=O)c2cccnc2)c(c1)N3CCN(CC3)S(=O)(=O)C | 3.698970004 | 5.0460288 |
| CHEMBL519312 | COc1c(Cl)cc(Cl)cc1C(=O)Nc2ccc(Br)cc2 | 3.698970004 | 4.3031783 |
| CHEMBL3799294 | Cc1nnc2c3c(c4ccccc4)c(c5ccccc5)n(c6cccc(Cl)c6)c3ncn12 | 3.823908741 | 3.7699071 |
| CHEMBL3798959 | Cc1cc(C)n(n1)c2ncnc3c2c(c4ccccc4)c(c5ccccc5)n3C6CCCCC6 | 3.823908741 | 3.7502913 |
| CHEMBL3798379 | Clc1cccc(c1)n2c3N=CNC(=O)c3c(c4ccccc4)c2c5ccccc5 | 3.823908741 | 3.5804606 |
| CHEMBL3800428 | Cc1ccc(Nc2ncnc3c2c(c4ccccc4)c(c5ccccc5)n3c6cccc(Cl)c6)cc1 | 3.823908741 | 3.5348032 |
| CHEMBL451080 | C[C@@]1(O)[C@H](O)[C@@](CO)(O[C@H]1N2C=CC(=NC2=O)N)N=[N+]=[N-] | 4 | 5.3448053 |
| CHEMBL497421 | CC(C)CCN1C(=C(C(=C(C2=CS(=O)(=O)c3ccccc3N2)C1=O)O)c4ccccc4)C | 4.060480747 | 5.4651797 |
| CHEMBL366330 | Cc1cc(C)nc(\N=C(/N)\NCCc2ccc(N)cc2)n1 | 4.214670165 | 4.5758889 |
| CHEMBL560524 | CCOC(=O)C1(CCCC1)NP(=O)(OC[C@]2(O[C@H]([C@H](O)[C@@H]2O)N3C=CC(=NC3=O)N)N=[N+]=[N-])Oc4ccccc4 | 4.251811973 | 4.9463411 |
| CHEMBL2011033 | CC(C)c1ccc(NC(=O)c2c(F)ccc(F)c2C)c(c1)N3CCN(CC3)c4cnccn4 | 4.26760624 | 4.8128088 |
| CHEMBL511764 | CC(C)CCc1nc(C2=NS(=O)(=O)c3ccccc3N2)c(O)c4ccccc14 | 4.292429824 | 5.3919045 |
| CHEMBL2387586 | OC(=O)[C@H](Cc1ccccc1)N2C(=S)S\C(=C/c3ccc(Oc4ccccc4)cc3)\C2=O | 4.294992041 | 5.3021916 |
| CHEMBL1090299 | OC(=O)c1cc2Cc3cc(cc(Cc4cc(cc(Cc5cc(cc(Cc(c1)c2O)c5O)C(=O)O)c4O)C(=O)O)c3O)C(=O)O | 4.301029996 | 4.8479753 |
| CHEMBL1090301 | OC(=O)c1cc2Cc3cc(cc(Cc4cc(cc(Cc5cc(cc(Cc(c1)c2OCc6ccccc6)c5OCc7ccccc7)C(=O)O)c4OCc8ccccc8)C(=O)O)c3OCc9ccccc9)C(=O)O | 4.301029996 | 4.1391613 |
| CHEMBL246324 | CCc1c(C)sc(NC(=O)CCc2ccccc2)c1C(=O)N | 4.301029996 | 5.0073273 |
| CHEMBL186869 | Cc1ccc(CC\N=C(/N)\Nc2nc(C)cc(C)n2)cc1 | 4.468521083 | 4.7338864 |
| CHEMBL513316 | CC(=O)N1C(C2=C(CC(C)(C)CC2=O)Nc3c(O)cccc13)c4ccccn4 | 4.494850022 | 4.6719907 |
| CHEMBL440562 | OC(=O)c1nc(nc(O)c1O)c2ccccc2 | 4.522878745 | 7.1175706 |
| CHEMBL2011018 | CC(C)c1ccc(NC(=O)c2cncnc2)c(c1)N3CCN(CC3)c4cnccn4 | 4.698970004 | 4.7537988 |
| CHEMBL1088783 | C[S+]([O-])CCNC(=O)c1c(F)cccc1OCC(=O)N[C@H](CO)Cc2ccccc2 | 4.698970004 | 6.5062767 |
| CHEMBL1092624 | OC[C@H](Cc1ccccc1)NC(=O)COc2cccc(F)c2C(=O)NCCC3CCCCC3 | 4.698970004 | 7.2623726 |
| CHEMBL524811 | OC1=C(C2=CS(=O)(=O)c3ccccc3N2)C(=O)N(Cc4ccccc4F)c5ccccc15 | 4.756961951 | 6.0768335 |
| CHEMBL1290319 | Nc1ncnc2c1ncn2Cc3cn(CP(=O)(O)O)nn3 | 4.795880017 | 4.7951629 |
| CHEMBL275938 | OC(=O)C1=CC(=C(c2ccc(O)c(c2)C(=O)O)c3ccc(O)c(c3)C(=O)O)C=CC1=O | 4.823908741 | 5.3656466 |
| CHEMBL3763814 | CC1(C)CC(=O)C2=C(C1)c3c(NC2c4oc(cc4)c5ccc(O)c(c5)C(=O)O)ccc6ccccc36 | 4.826813732 | 8.2258499 |
| CHEMBL3234967 | COc1ccc(cc1)c2cc3cc(OC)ccc3nc2C(O)c4ccccc4 | 4.849857838 | 4.9488082 |
| CHEMBL2387584 | OC(=O)[C@H](Cc1ccccc1)N2C(=S)S\C(=C/c3ccccc3Oc4ccccc4)\C2=O | 4.876148359 | 5.0218019 |
| CHEMBL259837 | COc1ccc(\C=C\C2=CN(CCC(C)C)C(=O)C(=C2O)C3=NS(=O)(=O)c4cc(NS(=O)(=O)C)ccc4N3)cc1 | 4.898871824 | 7.4873251 |
| CHEMBL3234966 | COc1ccc(cc1)c2cc3ccccc3nc2C(O)c4ccccc4 | 4.900664722 | 4.9269842 |
| CHEMBL2011003 | O=C(Nc1ccc(cc1N2CCN(CC2)c3cnccn3)C4CC4)c5cccnc5 | 4.920818754 | 4.8978162 |
| CHEMBL2164631 | OC(=O)c1cccc(N\N=C\c2cc(C(=O)NCCCCc3ccccc3)c4ccccc4n2)c1 | 4.958607315 | 4.5503988 |
| CHEMBL1097813 | CCC[C@@]1(OCCc2c1[nH]c3c(C)ccc(C#N)c23)[C@H](NC(=O)OC(C)C)C(=O)O | 4.958607315 | 5.4336213 |
| CHEMBL1929353 | OC(=O)C1=C2Sc3ccc(OC4CCCCC4)cc3N2C(=O)C(=C1)c5ccccc5 | 4.958607315 | 5.2427718 |
| CHEMBL2398072 | FCC(CF)NS(=O)(=O)c1ccc(nc1)c2c(C#N)c3ccc(cc3n2C4CCC4)C5CC5 | 5 | 4.9540306 |
| CHEMBL1091238 | OCCNc1ccc2C(=O)N(c3cccc(Cl)c3)c4nccn4c2c1 | 5 | 4.9432834 |
| CHEMBL2435788 | COc1ccc(O)c(CNc2ccc(c(OC)c2)c3ocnc3)c1 | 5.002176919 | 5.2906089 |
| CHEMBL2170015 | CC1(C)COC(=O)N1C2CCN(CC2)C(=O)c3nc4c(cc(cn4c3Cl)c5cocc5)C(F)(F)F | 5.045757491 | 4.9891238 |
| CHEMBL2387640 | COc1ccc(Oc2cccc(\C=C\3/SC(=S)N([C@@H](Cc4ccccc4)C(=O)O)C3=O)c2)cc1 | 5.080921908 | 6.9860171 |
| CHEMBL1091526 | O=C(Nc1ccncc1)c2cccc3C(=O)c4cccc(OCc5ccccc5)c4Nc23 | 5.096910013 | 4.7984499 |
| CHEMBL416461 | CSC(=O)N1[C@H]2CCN([C@@H]2C3(CCC3)C1=O)C(=O)[C@@H](NC(=O)OC(C)(C)C)C(C)C | 5.102372909 | 5.7726173 |
| CHEMBL2387577 | OC(=O)[C@H](Cc1ccccc1)N2C(=S)S\C(=C/c3cccc(Br)c3)\C2=O | 5.13076828 | 4.735057 |
| CHEMBL2011069 | Nc1cc(Cn2c(C(=O)O)c(C3=CC=CNC3=O)c4cc(ccc24)C5=CC=CC(=O)N5)ccn1 | 5.15490196 | 4.7811259 |
| CHEMBL1093221 | Cc1c(NC2CCN(Cc3ccccc3)CC2)ccc4C(=O)N(c5cccc(Cl)c5)c6nncn6c14 | 5.180456064 | 6.1926079 |
| CHEMBL2111998 | CCCCCCCCCCCCNC1=NC(=O)c2c(ncn2[C@@H]3C[C@H](O)[C@@H](CO)O3)C(=O)N1 | 5.189003436 | 3.7827248 |
| CHEMBL1077377 | OC(=O)c1nc(nc(O)c1O)c2ccsc2 | 5.211054273 | 5.191703 |
| CHEMBL388635 | OC(=O)c1ccccc1NC(=O)COc2ccc(F)cc2 | 5.283996656 | 5.3842629 |
| CHEMBL3798851 | C[C@H]1CCN2C[C@]34Cc5c([nH]c6c7C(=O)CC(C)(C)Oc7ccc56)C(C)(C)[C@@H]3C[C@]12C(=O)N4 | 5.292429824 | 5.4610249 |
| CHEMBL1087366 | CCC(C)C1(CC(=O)O)OCCc2c1[nH]c3c(C)ccc(C#N)c23 | 5.30980392 | 5.5739358 |
| CHEMBL2385619 | Nc1ncnc2c1ncn2[C@@H]3O[C@H](CCP(=O)(O)O)[C@@H](O)[C@H]3F | 5.337242168 | 5.5644296 |
| CHEMBL2092974 | NC1=NC(=O)C2C(N=CN2[C@H]3O[C@H](CO)[C@@H](O)[C@H]3O)C(=O)N1 | 5.346787486 | 6.1016706 |
| CHEMBL3786159 | Cc1nc(NC(=O)C2CCCC2)sc1c3ccc(Cl)c(c3)S(=O)(=O)NCCO | 5.356547324 | 4.8561041 |
| CHEMBL1090297 | CCCCOc1c2Cc3cc(cc(Cc4cc(cc(Cc5cc(cc(Cc1cc(c2)C(=O)N[C@@H](CCC(=O)O)C(=O)O)c5OCCCC)C(=O)N[C@@H](CCC(=O)O)C(=O)O)c4OCCCC)C(=O)N[C@H](CCC(=O)O)C(=O)O)c3OCCCC)C(=O)N[C@@H](CCC(=O)O)C(=O)O | 5.387216143 | 5.5013631 |
| CHEMBL2387638 | OC(=O)[C@H](Cc1ccccc1)N2C(=S)S\C(=C/c3cccc(Oc4cccc(Cl)c4)c3)\C2=O | 5.387216143 | 5.160913 |
| CHEMBL1784247 | CNC(=O)c1ccc(c(COc2ccc(cc2)n3nc(\C=C(/C)\C(=O)O)cc3C4CCCCC4)c1)c5ccc(Cl)cc5 | 5.394694954 | 7.0110314 |
| CHEMBL2011049 | CC(C)c1ccc(NC(=O)c2cccnc2)c(c1)N3CCN(CC3)c4nc(C)cs4 | 5.408935393 | 5.2821612 |
| CHEMBL2435785 | COc1ccc(CNc2ccc(c(OC)c2)c3ocnc3)cn1 | 5.488116639 | 5.7839251 |
| CHEMBL2011024 | CC(C)c1ccc(NC(=O)c2sc(Br)nc2C)c(c1)N3CCN(CC3)c4cnccn4 | 5.522878745 | 5.8151927 |
| CHEMBL1088812 | OC[C@H](Cc1ccccc1)NC(=O)COc2cccc(F)c2C(=O)N3CCCCCC3 | 5.585026652 | 5.3147183 |
| CHEMBL1091991 | CCCN(CC1CC1)C(=O)c2c(F)cccc2OCC(=O)N[C@H](CO)Cc3ccccc3 | 5.585026652 | 5.3155024 |
| CHEMBL1092686 | CN1C(=O)N(C(=O)c2ccc(NCCO)c(C)c12)c3cccc(Cl)c3 | 5.619788758 | 5.9655062 |
| CHEMBL508436 | OC1=C(C2=CS(=O)(=O)c3ccccc3N2)C(=O)N(CCC4CC4)c5ccc(Cl)cc15 | 5.619788758 | 6.1383415 |
| CHEMBL219795 | COC[C@@H]1C[C@](CC(C)C)(N([C@H]1c2nccs2)C(=O)c3ccc(c(OC)c3)C(C)(C)C)C(=O)O | 5.638272164 | 6.1135031 |
| CHEMBL2435798 | COc1cc(NCc2ccc(C)s2)ccc1c3ocnc3 | 5.688246139 | 5.7046779 |
| CHEMBL3637932 | CC(C)(C)OC(=O)N1CCN(CC1)c2nccc(n2)c3ccc(s3)c4ccc(Cl)cc4 | 5.698970004 | 5.9981174 |
| CHEMBL223720 | CN(C)CC1COc2ccc3nc4NCC(CN(C)C)Cn4c3c2C1 | 5.698970004 | 5.4857475 |
| CHEMBL568402 | C[C@@]1(Cc2ccc(F)cc2)CN(C3CCCCC3)C(=O)C(=C1O)C4=NS(=O)(=O)c5cc(NS(=O)(=O)C)ccc5N4 | 5.698970004 | 4.0630958 |
| CHEMBL258138 | CCC(Oc1ccc2NC(=NS(=O)(=O)c2c1)C3=C(O)C(=NN(CCC(C)C)C3=O)c4cccs4)C(=O)N | 5.809668302 | 7.0135756 |
| CHEMBL1090296 | CCCCOc1c2Cc3cc(cc(Cc4cc(cc(Cc5cc(cc(Cc1cc(c2)C(=O)N[C@@H](CC(=O)O)C(=O)O)c5OCCCC)C(=O)N[C@@H](CC(=O)O)C(=O)O)c4OCCCC)C(=O)N[C@H](CC(=O)O)C(=O)O)c3OCCC)C(=O)N[C@@H](CC(=O)O)C(=O)O | 5.823908741 | 5.7446852 |
| CHEMBL512356 | CCOc1cccc2C(=NS(=O)(=O)c12)C3=C(O)[C@@H](N(CCC(C)(C)C)C3=O)C(C)(C)C | 5.823908741 | 5.4163232 |
| CHEMBL180292 | CC(NN1C(=O)C(=C(O)c2ccccc12)C3=NS(=O)(=O)c4ccccc4N3)c5ccccc5 | 5.826813732 | 6.3010781 |
| CHEMBL2371795 | CCC[C@H](NC(=O)[C@@H]1C2CCCC2CN1C(=O)[C@@H](NC(=O)[C@@H](NC(=O)c3cnccn3)[C@@H](C)CC)C(C)(C)C)C(=O)C(=O)NC4CC4 | 5.841637508 | 5.9138397 |
| CHEMBL430718 | OC(=O)[C@H](Cc1ccccc1)N(Cc2cccc(Cl)c2)C(=O)c3ccc(Cl)cc3Cl | 5.853871964 | 6.1715335 |
| CHEMBL522404 | CC(C)CCN1C(=O)C(=C(O)c2cccnc12)C3=CS(=O)(=O)c4ccccc4N3 | 5.853871964 | 5.7823753 |
| CHEMBL2435795 | COc1cc(ccc1c2ocnc2)\N=C\c3ccc(F)cc3 | 5.928117993 | 5.6550195 |
| CHEMBL373376 | CC(C)C[C@]1(C[C@@H]([C@@H](N1C(=O)c2ccc(cc2)C(C)(C)C)c3cccs3)C(=O)O)C(=O)O | 5.958607315 | 6.5244631 |
| CHEMBL565444 | C[C@@]1(CN(Cc2ccc(F)cc2)C(=O)C(=C1O)C3=NS(=O)(=O)c4cc(NS(=O)(=O)C)ccc4N3)c5ccccc5 | 5.958607315 | 6.650539 |
| CHEMBL512956 | CC(=O)N1[C@H](C2=C(CC(C)(C)CC2=O)Nc3ccccc13)c4ccc(OCc5ccccc5)c(Cl)c4 | 6 | 6.2369422 |
| CHEMBL375806 | COc1cc(ccc1C(C)(C)C)C(=O)N2[C@H]([C@H](CO)C[C@@]2(CC(C)C)C(=O)O)c3nccs3 | 6 | 5.4835978 |
| CHEMBL566281 | CC(C)(C)CC[C@]1(C)CN(C2CCCCC2)C(=O)C(=C1O)C3=NS(=O)(=O)c4cc(NS(=O)(=O)C)ccc4N3 | 6.040958608 | 6.5836878 |
| CHEMBL591090 | CC(NC(=O)c1ccc2c(c1)nc(c3cocc3)n2C4CCCCC4)C(=O)Nc5cccc(\C=C\C(=O)O)c5 | 6.045757491 | 5.8253607 |
| CHEMBL2403224 | Fc1ccc(CN2C=NC(=O)c3cc(Oc4ncccc4Br)ccc23)c(F)c1 | 6.065501549 | 5.7686904 |
| CHEMBL284328 | [Br-].CC[n+]1c(c2ccccc2)c3cc(N)ccc3c4ccc(N)cc14 | 6.091514981 | 4.9501515 |
| CHEMBL199603 | CCC[C@H](NC(=O)[C@@H]1C[C@@H]2CN1C(=O)[C@@H](NC(=O)Cc3cccc(OCCCO2)c3)C(C)(C)C)C(=O)C(=O)NCC(=O)N[C@H](CN(C)C)c4ccccc4 | 6.15490196 | 6.545506 |
| CHEMBL367430 | OC(=O)[C@H](Cc1c[nH]c2ccc(O)cc12)NC(=O)c3ccc4c(c3)nc(c5ccccn5)n4C6CCCCC6 | 6.161150909 | 7.2982649 |
| CHEMBL180706 | OC1=C(C2=NS(=O)(=O)c3ccccc3N2)C(=O)N(NCc4nccs4)c5ccccc15 | 6.171340103 | 6.5928418 |
| CHEMBL1778751 | Cc1cc(cnc1N)c2c(C3CCCCC3)c4ccc(cc4n2C)C(=O)NC(C)(C)C(=O)Nc5ccc(\C=C\C(=O)O)cc5 | 6.202040356 | 6.8629972 |
| CHEMBL446645 | CS(=O)(=O)Nc1ccc2NC(=NS(=O)(=O)c2c1)C3=C(O)[C@H]4CCC[C@H]4N(Cc5ccc(cc5)C(F)(F)F)C3=O | 6.207608311 | 7.564837 |
| CHEMBL223482 | C[C@@]1(F)[C@H](O)[C@@H](CO)O[C@H]1N2C=CC(=NC2=O)N | 6.22184875 | 6.7205195 |
| CHEMBL388519 | COc1cc(Cl)ccc1OCC(=O)Nc2ccccc2C(=O)O | 6.244125144 | 5.5193032 |
| CHEMBL459652 | CC(C)[C@@H]1N(Cc2ccccc2)C(=O)C(=C1O)C3=NS(=O)(=O)c4c(OCC(=O)N)cccc34 | 6.283996656 | 6.2924412 |
| CHEMBL1778737 | Cn1c(c2cccc3[nH]ccc23)c(C4CCCCC4)c5ccc(cc15)C(=O)NC(C)(C)C(=O)Nc6ccc(\C=C\C(=O)O)cc6 | 6.301029996 | 6.010175 |
| CHEMBL2312036 | CCCCCCCC(=O)CCCCCC\C=C\[C@H](C(=O)N[C@@H](Cc1cccs1)C(=O)O)[C@@](O)(CC(=O)O)C(=O)O | 6.30980392 | 6.5509115 |
| CHEMBL1957271 | OC1=C(C2=NS(=O)(=O)c3ccccc3N2)C(=O)N(NC4=CCOC=C4)c5ccccc15 | 6.35753548 | 6.8094272 |
| CHEMBL246462 | CC1(CC=C2CCC2)C(=O)C(=C(O)c3ccccc13)C4=NS(=O)(=O)c5cc(NS(=O)(=O)C)ccc5N4 | 6.37161107 | 6.5697426 |
| CHEMBL1778736 | Cn1c(c2ccc3[nH]ccc3c2)c(C4CCCCC4)c5ccc(cc15)C(=O)NC(C)(C)C(=O)Nc6ccc(\C=C\C(=O)O)cc6 | 6.37675071 | 5.9946897 |
| CHEMBL252188 | CCCC1=NN(CCC(C)C)C(=O)C(=C1O)C2=NS(=O)(=O)c3cc(OCC(=O)N)ccc3N2 | 6.397940009 | 5.1145525 |
| CHEMBL601480 | CC[C@@H](NC(=O)c1ccc2c(c1)nc(c3cocc3)n2C4CCCCC4)C(=O)Nc5ccc(\C=C\C(=O)O)cc5 | 6.397940009 | 5.7174354 |
| CHEMBL526553 | CS(=O)(=O)Nc1ccc2NC(=CS(=O)(=O)c2c1)C3=C(O)c4ccc(Cl)cc4N(Cc5ccc(F)cc5)C3=O | 6.415668776 | 8.0458578 |
| CHEMBL541914 | Cl.CC(C)CCN1N=C(C(=C(C1=O)C2=NS(=O)(=O)c3cc(OCC(=O)N4CCNC4)ccc3N2)O)c5cccs5 | 6.468521083 | 6.6818104 |
| CHEMBL479978 | CCCCC1(CCCC)C(=O)C(=C(O)c2ccccc12)C3=NS(=O)(=O)c4cc(OCC(=O)N)ccc4N3 | 6.48148606 | 6.6820367 |
| CHEMBL1083895 | CC[C@H]1COB(O)[C@H]1NC(=O)[C@@H]2C[C@H](CN2C(=O)[C@@H](NC(=O)OC3CCCC3)C(C)(C)C)OC(=O)N4Cc5cccc(F)c5C4 | 6.48148606 | 6.7504976 |
| CHEMBL1778740 | Cn1c(c2cnc3ccccc3c2)c(C4CCCCC4)c5ccc(cc15)C(=O)NC(C)(C)C(=O)Nc6ccc(\C=C\C(=O)O)cc6 | 6.537602002 | 6.9195444 |
| CHEMBL401769 | CC(C)CCN1N=C(C(=C(C2=NS(=O)(=O)c3cc(ccc3N2)N4CCCS4(=O)=O)C1=O)O)c5cccs5 | 6.537602002 | 6.1695812 |
| CHEMBL255954 | CC(C)CCN1N=C(C(=C(C2=NS(=O)(=O)c3cc(ccc3N2)c4cnn[nH]4)C1=O)O)c5cccs5 | 6.537602002 | 6.682284 |
| CHEMBL590851 | OC(=O)\C=C\c1ccc(NC(=O)CNC(=O)c2ccc3c(c2)nc(c4cocc4)n3C5CCCCC5)cc1 | 6.552841969 | 6.9659841 |
| CHEMBL263598 | CC(=O)N[C@@H](CC(=O)O)C(=O)N[C@@H](CCC(=O)O)C(=O)N[C@@H](C(c1ccccc1)c2ccccc2)C(=O)N[C@@H](CCC(=O)O)C(=O)N[C@@H](CC3CCCCC3)C(=O)N[C@@H](CS)C(=O)NCc4ccccc4 | 6.602059991 | 6.7999181 |
| CHEMBL402191 | CC(C)CCN1N=C(C(=C(C1=O)C2=NS(=O)(=O)c3cc(CS(=O)(=O)C)ccc3N2)O)c4cccs4 | 6.619788758 | 6.9920655 |
| CHEMBL566485 | CC(C)CC[C@H]1CN(Cc2ccc(F)cc2)C(=O)C(=C1O)C3=NS(=O)(=O)c4cc(NS(=O)(=O)C)ccc4N3 | 6.657577319 | 7.2607671 |
| CHEMBL2392414 | COc1c(cc(cc1C(C)(C)C)N2C=CC(=O)NC2=O)c3ccc4C(=CCc4c3)C(C)(C)NS(=O)(=O)C | 6.66756154 | 8.1904728 |
| CHEMBL561465 | CC(C)CCN1C(=O)C(=C(O)c2ccccc12)C3=NS(=O)(=O)c4c(C)nn(C)c4N3 | 6.698970004 | 6.4984714 |
| CHEMBL411830 | CCC[C@H](NC(=O)[C@@H]1[C@H]2CCC[C@H]2CN1C(=O)[C@@H](NC(=O)[C@@H](NC(=O)c3ncc[nH]3)C4CCCCC4)C(C)(C)C)C(=O)C(=O)NC5CC5 | 6.698970004 | 7.7387565 |
| CHEMBL376104 | CC(=O)c1cc(Cl)c(F)cc1NCC(=O)Nc2ccccc2C(=O)O | 6.744727495 | 7.395268 |
| CHEMBL1778586 | CC(C)(NC(=O)c1ccc2c(C3CCCCC3)c([nH]c2c1)c4cocc4)C(=O)Nc5ccc(\C=C\C(=O)O)cc5 | 6.764471553 | 6.418111 |
| CHEMBL512251 | CC(C)C(=O)N1C(C2=C(CC(C)(C)CC2=O)Nc3c(O)cccc13)c4ccc(OCc5ccccc5)cc4Cl | 6.812479279 | 6.7382998 |
| CHEMBL457325 | Oc1c(nc(Cc2ccc(F)cc2)c3ccccc13)C4=NS(=O)(=O)c5c4cccc5c6cccnc6 | 6.823908741 | 6.8527046 |
| CHEMBL1778759 | Cn1c(c2cnc(N)nc2)c(C3CCCCC3)c4ccc(cc14)C(=O)NC(C)(C)C(=O)Nc5ccc(\C=C\C(=O)O)cc5 | 6.823908741 | 7.5389965 |
| CHEMBL1914869 | COC(=O)c1ccc2c(c1)c3CCCCc3n2CCCS(=O)(=O)N4C[C@@H](C)O[C@@H](C)C4 | 6.823908741 | 7.0367614 |
| CHEMBL497736 | OC1=C(C2=CS(=O)(=O)c3cc(F)ccc3N2)C(=O)N(Cc4ccc(F)cc4)c5ccc(F)cc15 | 6.829738285 | 6.1476332 |
| CHEMBL466468 | Cc1nc(cs1)C(=O)N2C(C3=C(CC(C)(C)CC3=O)Nc4c(O)cccc24)c5ccc(OCc6ccccc6)cc5F | 6.844663963 | 6.9471887 |
| CHEMBL1077321 | Oc1ccc2[nH]cc(C[C@H](NC(=O)c3ccc4c(c3)nc(c5ccccn5)n4C6CCCCC6)c7cscn7)c2c1 | 6.853871964 | 5.8312129 |
| CHEMBL371761 | CCCC(NC(=O)[C@@H]1C[C@@H]2CN1C(=O)[C@@H](NC(=O)Cc3cccc(OCCC(C)(C)O2)c3)C4CCCCC4)C(=O)C(=O)NCC(=O)N[C@H](C(=O)N(C)C)c5ccccc5 | 6.886056648 | 7.4884299 |
| CHEMBL2181642 | Cn1c(c2ccncc2)c(C3CCCC3)c4ccc(cc14)C(=O)NC5(CCC5)C(=O)Nc6ccc(\C=C\C(=O)O)cc6 | 6.886056648 | 6.7518143 |
| CHEMBL566473 | CC(=CC[C@H]1CN(Cc2ccc(F)cc2)C(=O)C(=C1O)C3=NS(=O)(=O)c4cc(NS(=O)(=O)C)ccc4N3)C | 6.886056648 | 7.3355326 |
| CHEMBL466920 | CC(C)C(=O)N1C(C2=C(CC(C)(C)CC2=O)Nc3c(O)cccc13)c4ccc(OCc5ccccc5)cc4F | 6.910094889 | 6.8379267 |
| CHEMBL254968 | CC(C)CCN1C(=O)C(=C(O)c2cccn12)C3=NS(=O)(=O)c4cc(ccc4N3)N(C)S(=O)(=O)C | 6.958607315 | 6.5699425 |
| CHEMBL1086643 | CC(C)CCN1C(=O)C(=C(O)c2ccccc12)C3=NS(=O)(=O)c4cc(ccc4N3)C(=O)N(C)C | 6.991399828 | 7.138872 |
| CHEMBL594318 | Cn1c(c2cocc2)c(C3CCCCC3)c4ccc(cc14)C(=O)N[C@@H](Cc5c[nH]c6ccc(O)cc56)c7cscn7 | 7 | 6.4396063 |
| CHEMBL2403317 | COc1ccnc(Oc2ccc3N(Cc4c(F)cc(F)cc4F)C=NC(=O)c3c2)c1C(F)(F)F | 7 | 6.5751861 |
| CHEMBL270643 | CNC(=O)COc1ccc2NC(=NS(=O)(=O)c2c1)C3=C(O)C(=NN(CCC(C)C)C3=O)c4cccs4 | 7.036212173 | 7.2455557 |
| CHEMBL605616 | C[C@@H](NC(=O)c1ccc2c(c1)nc(c3cocc3)n2C4CCCCC4)C(=O)Nc5ccc6oc(cc6c5)C(=O)O | 7.045757491 | 7.4278558 |
| CHEMBL2041670 | C[C@@H]1CC[C@H](CC1)C(=O)N(N(C)C)c2cc(C#CC(C)(C)C)sc2C(=O)O | 7.055517328 | 6.4719696 |
| CHEMBL2041669 | CC(C)N(C(=O)[C@@H]1CC[C@@H](C)CC1)c2cc(C#CC(C)(C)C)sc2C(=O)O | 7.065501549 | 7.0244652 |
| CHEMBL568422 | CC[C@@]1(CCC(C)C)CN(C2CCCC2)C(=O)C(=C1O)C3=NS(=O)(=O)c4cc(NS(=O)(=O)C)ccc4N3 | 7.075720714 | 7.1814637 |
| CHEMBL1087567 | Oc1ccc2[nH]cc(C[C@H](NC(=O)c3ccc4c(c3)nc(c5occc5)n4C6CCCCC6)c7cscn7)c2c1 | 7.096910013 | 6.5801916 |
| CHEMBL585891 | CS(=O)(=O)Nc1ccc2NC(=NS(=O)(=O)c2c1)C3=C(O)C[C@@H](N(Cc4ccc(F)cc4)C3=O)c5ccccc5 | 7.107905397 | 7.1517814 |
| CHEMBL367825 | OC1=C(C2=NS(=O)(=O)c3ccccc3N2)C(=O)N(NCc4cccc(Br)c4)c5ccccc15 | 7.124938737 | 7.1829849 |
| CHEMBL1778746 | Cc1ccc(nc1)c2c(C3CCCCC3)c4ccc(cc4n2C)C(=O)NC(C)(C)C(=O)Nc5ccc(\C=C\C(=O)O)cc5 | 7.148741651 | 6.8602372 |
| CHEMBL247083 | CC1(CCC2CCCC2)C(=O)C(=C(O)c3ccccc13)C4=NS(=O)(=O)c5cc(NS(=O)(=O)C)ccc5N4 | 7.15490196 | 7.0813628 |
| CHEMBL247288 | CC(C)(Cl)CCC1(C)C(=O)C(=C(O)c2ccccc12)C3=NS(=O)(=O)c4cc(NS(=O)(=O)C)ccc4N3 | 7.15490196 | 7.3704784 |
| CHEMBL501428 | Cc1cccc(n1)C(=O)N2C(C3=C(CC(C)(C)CC3=O)Nc4c(O)cccc24)c5ccc(OCc6ccccc6)cc5F | 7.15490196 | 6.7034784 |
| CHEMBL2347457 | CS(=O)(=O)NC(=O)c1c(C2=CC=CNC2=O)c3c(ccc4ccsc34)n1Cc5cc(F)ccc5F | 7.173925197 | 8.8071987 |
| CHEMBL567779 | CC(C)CC[C@@H]1CN(C2CCCC2)C(=O)C(=C1O)C3=NS(=O)(=O)c4cc(NS(=O)(=O)C)ccc4N3 | 7.251811973 | 7.0782467 |
| CHEMBL252398 | NC(=O)COc1ccc2NC(=NS(=O)(=O)c2c1)C3=C(O)C(=NN(CC4CCC4)C3=O)c5cccs5 | 7.259637311 | 6.6885886 |
| CHEMBL1651385 | CNC1COc2ccccc2c3c(C4CCCCC4)c5ccc(cc5n3C1)C(=O)O | 7.292429824 | 7.0769598 |
| CHEMBL1085436 | CC(C)CCN1C(=O)C(=C(O)c2ccccc12)C3=NS(=O)(=O)c4cc(C)ccc4N3 | 7.301029996 | 7.7906423 |
| CHEMBL1914888 | COC(=O)c1ccc2c(c1)c3CCCCc3n2CCCS(=O)(=O)c4ccccc4 | 7.301029996 | 8.3469585 |
| CHEMBL408675 | CC(C)CCN1N=C(C(=C(C1=O)C2=NS(=O)(=O)c3cc(NS(=O)(=O)C)ccc3N2)O)C4=CCCC4 | 7.30980392 | 7.2478159 |
| CHEMBL362909 | CCN(CC)C1CCN(CC1)C(=O)Cn2c(c3ccc(Cl)cc3)c(C4CCCCC4)c5ccc(cc25)C(=O)O | 7.318758763 | 7.2288996 |
| CHEMBL478017 | CC(C)CCN1N=C(N2CCCC2C)C(=C(C1=O)C3=NS(=O)(=O)c4cc(NS(=O)(=O)C)ccc4N3)O | 7.327902142 | 7.5362187 |
| CHEMBL2403637 | CO[C@]1(C[C@H](N(C1)C(=O)[C@@H](NC(=O)OC2CCCC2)C(C)(C)C)C(=O)N[C@@]3(C[C@H]3C=C)C(=O)NS(=O)(=O)C4CC4)c5ccccc5C | 7.356547324 | 7.0857224 |
| CHEMBL251790 | CC(C)CCN1N=C(C(=C(C2=Nc3ccc(OCC(=O)N)cc3S(=O)(=O)N2)C1=O)O)c4cncs4 | 7.408935393 | 6.9390067 |
| CHEMBL1085894 | NC(=O)\C=C\c1ccc2NC(=NS(=O)(=O)c2c1)C3=C(O)c4cc(F)ccc4N(CCC5CC5)C3=O | 7.408935393 | 8.0008377 |
| CHEMBL2347458 | OC(=O)c1c(C2=CC=CNC2=O)c3c4ccoc4ccc3n1Cc5cc(F)ccc5F | 7.468521083 | 7.9348759 |
| CHEMBL256324 | CS(=O)(=O)Nc1ccc2NC(=NS(=O)(=O)c2c1)C3=C(O)C(=NN(Cc4ccccn4)C3=O)c5cccs5 | 7.48148606 | 7.0419296 |
| CHEMBL2203893 | COc1ccc2cc3CCCCCO[C@H]4CCN(C4)C(=O)N[C@@H](C5CCCC5)C(=O)N6C[C@@H](C[C@H]6C(=O)N[C@@]7(C[C@H]7C=C)C(=O)NS(=O)(=O)C8CC8)Oc3nc2c1 | 7.508638306 | 8.3127184 |
| CHEMBL403642 | CC(C)CCN1N=C(C(=C(C2=NS(=O)(=O)c3cc(NS(=O)(=O)C(C)C)ccc3N2)C1=O)O)c4cccs4 | 7.522878745 | 6.7237731 |
| CHEMBL1082874 | COc1ccc2NC(=NS(=O)(=O)c2c1)C3=C(O)c4cc(F)ccc4N(CCC5CC5)C3=O | 7.619788758 | 7.9676578 |
| CHEMBL1088061 | COC(=O)Cc1ccc2NC(=NS(=O)(=O)c2c1)C3=C(O)c4ccccc4N(CCC(C)C)C3=O | 7.638272164 | 7.9496106 |
| CHEMBL1086917 | CC(C)(Oc1ccc2NC(=NS(=O)(=O)c2c1)C3=C(O)c4cc(F)ccc4N(CCC5CC5)C3=O)C(=O)O | 7.657577319 | 6.615931 |
| CHEMBL2390971 | COc1c(cc(cc1C(C)(C)C)N2C=CC(=O)NC2=O)c3ccc4c(CNS(=O)(=O)C)coc4c3 | 7.677780705 | 7.4682932 |
| CHEMBL504737 | CC1(C)CC2=C(C(N(C(=O)c3ncn[nH]3)c4cccc(O)c4N2)c5ccc(OCc6ccccc6)cc5F)C(=O)C1 | 7.677780705 | 7.0051199 |
| CHEMBL1651392 | CN1CCC(CC1)NC2COc3ccccc3c4c(C5CCCCC5)c6ccc(cc6n4C2)C(=O)O | 7.677780705 | 8.0999542 |
| CHEMBL1778758 | Cn1c(c2cnccn2)c(C3CCCCC3)c4ccc(cc14)C(=O)NC(C)(C)C(=O)Nc5ccc(\C=C\C(=O)O)cc5 | 7.698970004 | 7.7342014 |
| CHEMBL1651405 | CN(C)CCO[C@@H]1COc2ccccc2c3c(C4CCCCC4)c5ccc(cc5n3C1)C(=O)O | 7.721246399 | 7.3781346 |
| CHEMBL2312023 | CCCCCCC(=O)CCCCCC\C=C\[C@H](C(=O)N[C@@H](Cc1ccc(OCCC(C)C)cc1)C(=O)O)[C@@](O)(CC(=O)O)C(=O)O | 7.721246399 | 8.0493571 |
| CHEMBL256949 | CC(C)CCN1N=C(C(=C(C1=O)C2=NS(=O)(=O)c3cc(NS(=O)(=O)CCN)ccc3N2)O)c4cccs4 | 7.721246399 | 7.6431696 |
| CHEMBL2431463 | COc1c(cc(cc1C(C)(C)C)C2=CC=CNC2=O)C3CC3c4ccc(NS(=O)(=O)C)cc4 | 7.823908741 | 7.3290845 |
| CHEMBL1928118 | OC(=O)\C(=C\C(=O)c1cccc(OCc2cc(C#N)c(Cl)cc2Cl)c1)\O | 7.823908741 | 7.7949035 |
| CHEMBL2312012 | CCCCCCCC(=O)CCCCCC\C=C\[C@H](C(=O)N[C@@H](Cc1ccc2ccccc2c1)C(=O)O)[C@@](O)(CC(=O)O)C(=O)O | 7.853871964 | 8.0353092 |
| CHEMBL525553 | CC(C)CCN1N2CCCC2C(=C(C1=O)C3=NS(=O)(=O)c4cc(NS(=O)(=O)C)ccc4N3)O | 7.920818754 | 8.031076 |
| CHEMBL516376 | Cc1cc(CN2[C@@H]3CCC[C@@H]3C(=C(C2=O)C4=NS(=O)(=O)c5cc(NS(=O)(=O)C)ccc5N4)O)ccc1F | 8 | 7.5576659 |
| CHEMBL372357 | OC1=C(C(=O)N(CCC2CC2)c3ccc(F)cc13)C4=Nc5ccccc5S(=O)(=O)N4 | 8 | 7.5770921 |
| CHEMBL2312022 | CCCCCCCC(=O)CCCCCC\C=C\[C@H](C(=O)N[C@@H](Cc1ccc(cc1)c2cccnc2)C(=O)O)[C@@](O)(CC(=O)O)C(=O)O | 8 | 7.6287157 |
| CHEMBL258459 | CC(C)(C)CCN1N=C(C(=C(C1=O)C2=NS(=O)(=O)c3cc(NS(=O)(=O)C)ccc3N2)O)c4cccs4 | 8 | 7.2186705 |
| CHEMBL2347452 | Fc1ccc(F)c(Cn2c(C(=O)NS(=O)(=O)Cc3ccccc3)c(C4=CC=CNC4=O)c5cc(Cl)ccc25)c1 | 8 | 7.7020489 |
| CHEMBL1672596 | C=C[C@@H]1C[C@]1(NC(=O)[C@@H]2C[C@@H]3CN2C(=O)[C@@H](NC(=O)OCCC\C=C\c4ccc5ccnc(O3)c5c4)C6CCOCC6)C(=O)NS(=O)(=O)C7CC7 | 8.045757491 | 7.9145053 |
| CHEMBL2431464 | COc1c(cc(cc1C(C)(C)C)C2=CC=CNC2=O)c3oc4ccc(NS(=O)(=O)C)cc4n3 | 8.045757491 | 8.1293171 |
| CHEMBL2011078 | Cc1ccccc1Cn2c(C(=O)O)c(C3=CC=CNC3=O)c4cc(ccc24)C(F)(F)F | 8.045757491 | 8.489043 |
| CHEMBL2347470 | CS(=O)(=O)NC(=O)c1c(C2=CC=CNC2=O)c3c4occc4ccc3n1Cc5ccccc5F | 8.096910013 | 8.1088648 |
| CHEMBL2402055 | CC(C)(C)c1cc(cc(C(=O)Nc2ccc(NS(=O)(=O)CCN3CCOCC3)cc2)c1O)N4CCC(=O)NC4=O | 8.15490196 | 7.7185021 |
| CHEMBL1956812 | COc1cccc(CP(=O)(O)[C@]2(C[C@H]2C=C)NC(=O)[C@@H]3C[C@H](CN3C(=O)[C@@H](NC(=O)OC4CCCC4)C(C)(C)C)Oc5cc(nc6cc(OC)ccc56)c7csc(NC(C)C)n7)c1 | 8.15490196 | 7.0123303 |
| CHEMBL1779114 | COc1ccc2c(O[C@@H]3C[C@H](N(C3)C(=O)[C@@H](NC(=O)OC4CCCC4)C(C)(C)C)C(=O)N[C@@]5(C[C@H]5C=C)P(=O)(O)Cc6ccccc6)cc(nc2c1)c7csc(NC(C)C)n7 | 8.15490196 | 7.9215669 |
| CHEMBL560922 | CC(C)CCN1C(=O)C(=C(O)c2ccccc12)C3=NS(=O)(=O)c4cc(OCC(=O)N)ccc4N3 | 8.22184875 | 8.1997415 |
| CHEMBL1779118 | COc1ccc2c(O[C@@H]3C[C@@H]4N(C3)C(=O)[C@H](CCCCCCC[C@@H]5C[C@]5(NC4=O)P(=O)(O)Cc6ccccc6)NC(=O)OC7CCCC7)cc(nc2c1)c8csc(NC(C)C)n8 | 8.22184875 | 8.1113912 |
| CHEMBL2347466 | CCS(=O)(=O)NC(=O)c1c(C2=CC=CNC2=O)c3c4occc4ccc3n1Cc5cc(F)ccc5F | 8.22184875 | 7.8689211 |
| CHEMBL3121324 | COc1ncc(cc1S(=O)(=O)Nc2ccc(F)cc2F)c3ccc4N=C(N)N(C(=O)c4c3)c5ccccc5 | 8.299988938 | 8.5612524 |
| CHEMBL1082873 | OC1=C(C2=NS(=O)(=O)c3cc(O)ccc3N2)C(=O)N(CCC4CC4)c5ccc(F)cc15 | 8.301029996 | 7.5590815 |
| CHEMBL565485 | CCC[C@H](NC(=O)[C@@H]1C[C@H](CN1C(=O)[C@@H](NC(=O)OCCN2CCCC2)C(C)(C)C)OC(=O)N3Cc4ccccc4C3)B5O[C@@H]6C[C@@H]7C[C@@H](C7(C)C)[C@]6(C)O5 | 8.301029996 | 7.9679631 |
| CHEMBL564554 | CC(C)CCN1C(=O)C(=C(O)c2sccc12)C3=NS(=O)(=O)c4cc(OCC(=O)N)ccc4N3 | 8.301029996 | 8.165874 |
| CHEMBL1672587 | CC(C)(C)[C@@H]1NC(=O)OCCC\C=C\c2ccc3ccnc(O[C@@H]4C[C@H](N(C4)C1=O)C(=O)N[C@@]5(C[C@H]5C=C)C(=O)NS(=O)(=O)C6CC6)c3c2 | 8.346787486 | 8.2387347 |
| CHEMBL2206959 | COc1cc2c3ccc(cc3nc4O[C@@H]5C[C@H](N(C5)C(=O)[C@@H](NC(=O)OCC(C)(C)CCCc1cc24)C6CCCC6)C(=O)N[C@@]7(C[C@H]7C=C)C(=O)NS(=O)(=O)C8CC8)C#N | 8.397940009 | 8.1020347 |
| CHEMBL289765 | COc1ccc2c(O[C@@H]3C[C@@H]4N(C3)C(=O)[C@H](CCCCC\C=C/[C@@H]5C[C@]5(NC4=O)C(=O)O)NC(=O)OC6CCCC6)cc(nc2c1)c7csc(N)n7 | 8.455931956 | 8.3240079 |
| CHEMBL2403730 | COc1ccc(cc1)c2ccc(cc2)[C@@]3(C[C@H](N(C3)C(=O)[C@@H](NC(=O)OC4CCCC4)C(C)(C)C)C(=O)N[C@@]5(C[C@H]5C=C)C(=O)NS(=O)(=O)C6CC6)OC | 8.522878745 | 8.5453583 |
| CHEMBL1956822 | COc1ccc2c(O[C@@H]3C[C@@H]4N(C3)C(=O)[C@H](CCCCCCC[C@@H]5C[C@]5(NC4=O)P(=O)(O)Cc6c(F)cccc6F)NC(=O)OC7CCCC7)cc(nc2c1Br)c8csc(NC(C)C)n8 | 8.522878745 | 8.0800997 |
| CHEMBL1672606 | COc1cc2ccnc3O[C@@H]4C[C@H](N(C4)C(=O)[C@@H](NC(=O)OCCCCCc1cc23)C(C)(C)C)C(=O)N[C@@]5(C[C@H]5C=C)C(=O)NS(=O)(=O)C6CC6 | 8.522878745 | 8.3083335 |
| CHEMBL2206984 | COc1cc2c3ccccc3nc4O[C@@H]5C[C@H](N(C5)C(=O)[C@@H](NC(=O)OCC(C)(C)CCCc1cc24)C6CCCC6)C(=O)N[C@@]7(C[C@H]7C=C)C(=O)NS(=O)(=O)C8CC8 | 8.522878745 | 8.0480792 |
| CHEMBL1938988 | CC(C)[C@H]1CC[C@@H]2[C@@H](C[C@H](N2C1=O)C(=O)N[C@@]3(C[C@H]3C=C)C(=O)NS(=O)(=O)C4CC4)Oc5cc(nc6c(Cl)c(OCCN7CCOCC7)ccc56)c8nc(cs8)C(C)C | 8.522878745 | 8.0389875 |
| CHEMBL43030 | COc1ccc2c(O[C@@H]3C[C@@H]4N(C3)C(=O)[C@H](CCCCC\C=C/[C@@H]5C[C@]5(NC4=O)C(=O)O)NC(=O)OC6CCCC6)cc(nc2c1)n7cccn7 | 8.657577319 | 9.0678465 |
| CHEMBL1672610 | CC1(C)CCCc2ccc3ccnc(O[C@@H]4C[C@H](N(C4)C(=O)[C@@H](NC(=O)OC1)C5CCCC5)C(=O)N[C@@]6(C[C@H]6C=C)C(=O)NS(=O)(=O)C7CC7)c3c2 | 8.698970004 | 8.1232956 |
| CHEMBL2203873 | CCOc1cc2O[C@@H]3C[C@H](N(C3)C(=O)[C@@H](NC(=O)OCC(C)(C)CCCc4cc2c(cc4OC)n1)C5CCCC5)C(=O)N[C@@]6(C[C@H]6C=C)C(=O)NS(=O)(=O)C7CC7 | 8.698970004 | 8.458104 |
| CHEMBL568754 | CCC[C@H](NC(=O)[C@@H]1C[C@H](CN1C(=O)[C@@H](NC(=O)OC(C)(C)C)C2CCCCC2)OC(=O)N3Cc4ccccc4C3)B(O)O | 8.698970004 | 8.90619 |
| CHEMBL1672598 | CC(C)(C)[C@@H]1NC(=O)OCCCCCc2cc(Br)c3ccnc(O[C@@H]4C[C@H](N(C4)C1=O)C(=O)N[C@@]5(C[C@H]5C=C)C(=O)NS(=O)(=O)C6CC6)c3c2 | 8.698970004 | 7.9489798 |
| CHEMBL1672608 | COc1cc2ccnc3O[C@@H]4C[C@H](N(C4)C(=O)[C@@H](NC(=O)OCCCCCc1cc23)C5CCCC5)C(=O)N[C@@]6(C[C@H]6C=C)C(=O)NS(=O)(=O)C7CC7 | 8.698970004 | 7.9023428 |
| CHEMBL499639 | CS(=O)(=O)Nc1ccc2NC(=CS(=O)(=O)c2c1)C3=C(O)c4ccccc4N(Cc5ccc(F)cc5)C3=O | 8.698970004 | 8.0080537 |
| CHEMBL2403736 | CO[C@]1(C[C@H](N(C1)C(=O)[C@@H](NC(=O)OC2CCCC2)C(C)(C)C)C(=O)N[C@@]3(C[C@H]3C=C)C(=O)NS(=O)(=O)C4CC4)c5ccc(cc5)c6nccs6 | 8.958607315 | 8.2215891 |
| CHEMBL2431366 | COc1c(\C=C\c2ccc(NS(=O)(=O)C)cc2)cc(cc1C(C)(C)C)C3=CC(=CNC3=O)Cl | 9 | 8.3905869 |
| CHEMBL2431465 | COc1c(cc(cc1C(C)(C)C)C2=CC=CNC2=O)C3=Cc4ccc(NS(=O)(=O)C)cc4C(=O)O3 | 9 | 8.5993047 |
| CHEMBL2431364 | COc1c(\C=C\c2ccc(NS(=O)(=O)C)cc2)cc(cc1C(C)(C)C)C3=NC=NNC3=O | 9 | 8.455601 |
| CHEMBL1092581 | CNC(=O)c1c(oc2cc(N(CCO)S(=O)(=O)C)c(cc12)C3CC3)c4ccc(F)cc4 | 9.096910013 | 6.4324795 |
| CHEMBL3121334 | COc1ncc(cc1NS(=O)(=O)c2ccc(F)cc2F)c3ccc4N=C(N)N(C(=O)c4c3)c5ccccc5 | 9.299988938 | 8.6637973 |

**Supplementary Table S4.** Actual and predicted pIC_50_ of 12 independent validation data set of peptides

| **AVPdb id** | **Sequence** | **Length** | **Actual pIC50** | **Predicted pIC50** |
| --- | --- | --- | --- | --- |
| AVP0173 | KGSVVIVGRIILSGRK | 16 | 5.244125144 | 4.6689246 |
| AVP0174 | VRLGSISVIGIVRGKK | 16 | 3.863279433 | 4.7993581 |
| AVP0616 | SWLRRIWRWICKVLSRFK | 18 | 6.050609993 | 5.4219168 |
| AVP0620 | LRDIWDWICEVLSDFK | 16 | 4.568636236 | 5.1774578 |
| AVP0653 | RIGRNPSQVGD | 11 | 3.54515514 | 3.5520865 |
| AVP1182 | MANAGLQLLGFILA | 14 | 4.602059991 | 4.5761761 |
| AVP1185 | MANAGLQLLGFILAFLGWIGAI | 22 | 5.397940009 | 4.6344116 |
| AVP1188 | AGALMFAWLLLGLQGIFN | 18 | 4.602059991 | 5.2638753 |
| AVP1212 | GELGRLVYLLDGPGYDPI | 18 | 7.744727495 | 6.4533429 |
| AVP1223 | LLDCWVRLGRYLLRRLKT | 18 | 5 | 4.9508584 |
| AVP1506 | SWLRDIWDWLCEVLSDFK | 18 | 5.920818754 | 5.6843673 |
| AVP1579 | FWFTLIKTQAKQPARYRRFC | 20 | 5.522878745 | 5.1390753 |

**Supplementary Table S5.** The actual and predicted pIC50 of the decoy sets

| **S. no** | **ZINC_IDS** | **pIC50** |
| --- | --- | --- |
| 1 | ZINC79178904 | 5.5244997 |
| 2 | ZINC95448002 | 6.3637459 |
| 3 | ZINC18214759 | 4.7430798 |
| 4 | ZINC90154538 | 4.0238011 |
| 5 | ZINC60168732 | 4.0603194 |
| 6 | ZINC72419262 | 5.0642259 |
| 7 | ZINC62323855 | 5.2504214 |
| 8 | ZINC20583675 | 5.2252261 |
| 9 | ZINC23989439 | 4.4889573 |
| 10 | ZINC11915525 | 4.6232445 |
| 11 | ZINC22745711 | 5.2381778 |
| 12 | ZINC09129076 | 5.830482 |
| 13 | ZINC40798554 | 4.3785171 |
| 14 | ZINC22851847 | 5.8752733 |
| 15 | ZINC12998796 | 5.0591263 |
| 16 | ZINC40170002 | 4.1883852 |
| 17 | ZINC22315153 | 7.1306272 |
| 18 | ZINC10058925 | 6.3885997 |
| 19 | ZINC14794019 | 5.6262058 |
| 20 | ZINC72089884 | 5.8829635 |
| 21 | ZINC09090168 | 5.1071869 |
| 22 | ZINC20007610 | 4.7775073 |
| 23 | ZINC27988761 | 4.9972755 |
| 24 | ZINC34037183 | 4.608473 |
| 25 | ZINC69636187 | 6.2675847 |
| 26 | ZINC95369254 | 6.1134824 |
| 27 | ZINC72072487 | 6.9194231 |
| 28 | ZINC08828720 | 6.2469283 |
| 29 | ZINC33326700 | 4.4988052 |
| 30 | ZINC12682827 | 5.1293628 |
| 31 | ZINC02805055 | 5.5264977 |
| 32 | ZINC13629335 | 4.6122007 |
| 33 | ZINC92535355 | 5.4417751 |
| 34 | ZINC16971975 | 4.9305187 |
| 35 | ZINC08610596 | 4.4457407 |
| 36 | ZINC59485826 | 4.194594 |
| 37 | ZINC15084783 | 4.3833885 |
| 38 | ZINC36558957 | 5.6884975 |
| 39 | ZINC07467050 | 6.184646 |
| 40 | ZINC58961272 | 4.8615642 |
| 41 | ZINC02238655 | 4.4599202 |
| 42 | ZINC89969541 | 6.1168621 |
| 43 | ZINC02925801 | 5.1522516 |
| 44 | ZINC65614938 | 5.1235177 |
| 45 | ZINC09864473 | 5.2429361 |
| 46 | ZINC33139442 | 5.485591 |
| 47 | ZINC13561638 | 5.4381995 |
| 48 | ZINC09629877 | 5.6924205 |
| 49 | ZINC20239504 | 5.5408649 |
| 50 | ZINC09337172 | 4.623538 |
| 51 | ZINC09405901 | 5.3959273 |
| 52 | ZINC41091026 | 6.6699349 |
| 53 | ZINC95116512 | 5.6540695 |
| 54 | ZINC09927377 | 5.4583943 |
| 55 | ZINC08854390 | 4.6760097 |
| 56 | ZINC34048998 | 5.1452026 |
| 57 | ZINC09359303 | 5.0166576 |
| 58 | ZINC03020798 | 5.6589405 |
| 59 | ZINC08757163 | 5.4091282 |
| 60 | ZINC36597378 | 5.3407775 |
| 61 | ZINC80507887 | 4.978012 |
| 62 | ZINC05433868 | 5.9979931 |
| 63 | ZINC72320267 | 5.2785618 |
| 64 | ZINC72079344 | 6.4498695 |
| 65 | ZINC09583126 | 4.1703084 |
| 66 | ZINC10219058 | 5.2844727 |
| 67 | ZINC09378613 | 4.7087882 |
| 68 | ZINC19852371 | 4.376026 |
| 69 | ZINC09116201 | 7.1395629 |
| 70 | ZINC21374449 | 5.8633865 |
| 71 | ZINC12892082 | 4.2663623 |
| 72 | ZINC36738311 | 4.7768769 |
| 73 | ZINC05440876 | 5.8176307 |
| 74 | ZINC72029186 | 4.257909 |
| 75 | ZINC12693457 | 5.7788963 |
| 76 | ZINC10013042 | 4.9795275 |
| 77 | ZINC02716213 | 6.0467656 |
| 78 | ZINC49649294 | 6.5091168 |
| 79 | ZINC36625093 | 6.0764045 |
| 80 | ZINC40387095 | 5.3060161 |
| 81 | ZINC39791496 | 5.8727823 |
| 82 | ZINC03267291 | 5.4464392 |
| 83 | ZINC14541023 | 5.0455536 |
| 84 | ZINC04176416 | 5.2486212 |
| 85 | ZINC32885706 | 7.1309771 |
| 86 | ZINC46141425 | 6.1107852 |
| 87 | ZINC09546054 | 5.1371811 |
| 88 | ZINC08719776 | 4.6415791 |
| 89 | ZINC20578581 | 3.8682435 |
| 90 | ZINC16543134 | 4.675939 |
| 91 | ZINC08589869 | 5.6634543 |
| 92 | ZINC37601951 | 6.0371415 |
| 93 | ZINC02997406 | 5.4872145 |
| 94 | ZINC09224275 | 6.051617 |
| 95 | ZINC12009633 | 6.0854556 |
| 96 | ZINC13134235 | 4.3243237 |
| 97 | ZINC13509491 | 4.3204559 |
| 98 | ZINC67238571 | 4.303168 |
| 99 | ZINC09775418 | 5.4646184 |
| 100 | ZINC20478125 | 4.9911857 |
| 101 | ZINC85649691 | 4.3980281 |
| 102 | ZINC02958736 | 5.1344925 |
| 103 | ZINC72085108 | 5.1468191 |
| 104 | ZINC21993820 | 5.1520215 |
| 105 | ZINC12528228 | 5.1822698 |
| 106 | ZINC02840578 | 5.0372771 |
| 107 | ZINC20182359 | 4.8759836 |
| 108 | ZINC05942083 | 4.5219757 |
| 109 | ZINC72321217 | 5.573068 |
| 110 | ZINC14360048 | 5.9082467 |
| 111 | ZINC33088325 | 4.6837919 |
| 112 | ZINC11447320 | 4.9323101 |
| 113 | ZINC20751914 | 6.8588429 |
| 114 | ZINC16475515 | 5.0643722 |
| 115 | ZINC40154855 | 5.0113388 |
| 116 | ZINC67166572 | 5.1623805 |
| 117 | ZINC94091121 | 5.1264302 |
| 118 | ZINC83411090 | 4.8298979 |
| 119 | ZINC12746455 | 4.2047977 |
| 120 | ZINC03241702 | 5.0083069 |
| 121 | ZINC21734848 | 5.1301151 |
| 122 | ZINC14097878 | 5.7900363 |
| 123 | ZINC03118276 | 4.9944562 |
| 124 | ZINC09621267 | 4.9410702 |
| 125 | ZINC20150312 | 4.9041634 |
| 126 | ZINC09467516 | 4.4769084 |
| 127 | ZINC01023951 | 5.4994819 |
| 128 | ZINC54617050 | 5.1677626 |
| 129 | ZINC21526254 | 4.9837392 |
| 130 | ZINC22402582 | 6.4138605 |
| 131 | ZINC12855711 | 5.3186518 |
| 132 | ZINC64952141 | 5.8351116 |
| 133 | ZINC15829935 | 4.9936109 |
| 134 | ZINC09445189 | 5.7251762 |
| 135 | ZINC02108285 | 5.2477312 |
| 136 | ZINC10775767 | 5.5398885 |
| 137 | ZINC58075035 | 5.2618475 |
| 138 | ZINC77926484 | 6.5336567 |
| 139 | ZINC59265410 | 4.0724044 |
| 140 | ZINC15777874 | 5.4743786 |
| 141 | ZINC02780331 | 4.7544317 |
| 142 | ZINC23873155 | 3.7758561 |
| 143 | ZINC95479498 | 4.8488273 |
| 144 | ZINC15412132 | 6.0456953 |
| 145 | ZINC02638675 | 5.0761995 |
| 146 | ZINC71810291 | 5.1158084 |
| 147 | ZINC40755901 | 4.7553014 |
| 148 | ZINC15678564 | 6.2431992 |
| 149 | ZINC09818823 | 4.9447514 |
| 150 | ZINC41027052 | 4.9440016 |
| 151 | ZINC71768619 | 5.6573642 |
| 152 | ZINC12727369 | 3.3906034 |
| 153 | ZINC03273270 | 5.452409 |
| 154 | ZINC23570449 | 5.7073158 |
| 155 | ZINC16730130 | 5.9547787 |
| 156 | ZINC06604907 | 4.3859817 |
| 157 | ZINC81512577 | 4.5142228 |
| 158 | ZINC19293228 | 5.7521419 |
| 159 | ZINC12818921 | 4.2537076 |
| 160 | ZINC48413514 | 4.9277993 |
| 161 | ZINC69636184 | 5.8984172 |
| 162 | ZINC07159456 | 4.2389893 |
| 163 | ZINC14161200 | 5.0237231 |
| 164 | ZINC09144700 | 4.0185586 |
| 165 | ZINC03640355 | 5.305838 |
| 166 | ZINC08604410 | 4.7488573 |
| 167 | ZINC69901777 | 5.1384107 |
| 168 | ZINC85877708 | 6.5388118 |
| 169 | ZINC33435894 | 5.9765397 |
| 170 | ZINC36359942 | 4.8516326 |
| 171 | ZINC64605863 | 4.9460997 |
| 172 | ZINC09914573 | 5.4375667 |
| 173 | ZINC08934947 | 3.774017 |
| 174 | ZINC10149437 | 5.3970008 |
| 175 | ZINC08719773 | 4.6570717 |
| 176 | ZINC74061597 | 4.5303113 |
| 177 | ZINC09820433 | 4.9781822 |
| 178 | ZINC89923975 | 5.53404 |
| 179 | ZINC04496118 | 5.2469155 |
| 180 | ZINC41006932 | 4.9850437 |
| 181 | ZINC71982026 | 4.9652204 |
| 182 | ZINC20134503 | 5.4512618 |
| 183 | ZINC22014627 | 4.7926813 |
| 184 | ZINC10119409 | 5.8584311 |
| 185 | ZINC72096541 | 5.4218451 |
| 186 | ZINC03604192 | 4.9061148 |
| 187 | ZINC91790054 | 5.2383205 |
| 188 | ZINC64991345 | 3.9733837 |
| 189 | ZINC71846163 | 4.5237768 |
| 190 | ZINC78659852 | 4.580344 |
| 191 | ZINC47163862 | 6.0132322 |
| 192 | ZINC71853691 | 4.2592107 |
| 193 | ZINC71933875 | 5.481776 |
| 194 | ZINC00998086 | 5.6248854 |
| 195 | ZINC06931128 | 5.0714767 |
| 196 | ZINC17334013 | 4.8077812 |
| 197 | ZINC11855917 | 5.4364374 |
| 198 | ZINC33270933 | 4.2235822 |
| 199 | ZINC21247753 | 4.7594398 |
| 200 | ZINC13108732 | 4.0934765 |
| 201 | ZINC79020903 | 5.6413539 |
| 202 | ZINC00892085 | 4.3933983 |
| 203 | ZINC70973672 | 4.2180068 |
| 204 | ZINC36752412 | 5.5419708 |
| 205 | ZINC11854832 | 5.6719745 |
| 206 | ZINC06767102 | 5.5810734 |
| 207 | ZINC09509287 | 5.1506132 |
| 208 | ZINC46900519 | 5.3345015 |
| 209 | ZINC21127264 | 4.855007 |
| 210 | ZINC39929726 | 4.4694604 |
| 211 | ZINC36360022 | 5.0863771 |
| 212 | ZINC58835037 | 4.811471 |
| 213 | ZINC09818804 | 4.8100502 |
| 214 | ZINC03227651 | 4.8837344 |
| 215 | ZINC71939973 | 4.3229842 |
| 216 | ZINC22508386 | 4.3448144 |
| 217 | ZINC09491561 | 5.1260375 |
| 218 | ZINC40760984 | 4.4409593 |
| 219 | ZINC02626180 | 4.829518 |
| 220 | ZINC02060682 | 6.3436283 |
| 221 | ZINC19183903 | 5.0006863 |
| 222 | ZINC58899516 | 5.4596988 |
| 223 | ZINC12950837 | 5.9193789 |
| 224 | ZINC09464132 | 5.225143 |
| 225 | ZINC35311599 | 4.7926757 |
| 226 | ZINC72228903 | 5.2524588 |
| 227 | ZINC40168289 | 4.0181942 |
| 228 | ZINC57524324 | 4.101891 |
| 229 | ZINC64219777 | 5.1127041 |
| 230 | ZINC09263067 | 5.9598869 |
| 231 | ZINC11444457 | 5.2121474 |
| 232 | ZINC11471683 | 4.5517961 |
| 233 | ZINC21651883 | 5.6436088 |
| 234 | ZINC14965003 | 5.7839834 |
| 235 | ZINC20633954 | 5.737583 |
| 236 | ZINC38864453 | 6.0069702 |
| 237 | ZINC36806295 | 4.3460044 |
| 238 | ZINC01194984 | 4.1569436 |
| 239 | ZINC01124077 | 5.5756979 |
| 240 | ZINC19742372 | 6.7118856 |
| 241 | ZINC08903543 | 4.2787755 |
| 242 | ZINC70055831 | 5.358932 |
| 243 | ZINC36358849 | 6.0936813 |
| 244 | ZINC13494649 | 4.8273187 |
| 245 | ZINC09261072 | 5.4991591 |
| 246 | ZINC06385939 | 4.7833627 |
| 247 | ZINC59591450 | 4.9531828 |
| 248 | ZINC04969995 | 4.8021016 |
| 249 | ZINC13503904 | 4.9865649 |
| 250 | ZINC32529402 | 6.4111725 |
| 251 | ZINC12543607 | 4.7486196 |
| 252 | ZINC09206136 | 4.6385453 |
| 253 | ZINC77298801 | 4.3557537 |
| 254 | ZINC09171318 | 4.6889717 |
| 255 | ZINC91190689 | 5.5081309 |
| 256 | ZINC13401819 | 5.6402067 |
| 257 | ZINC17051910 | 3.7981013 |
| 258 | ZINC77866667 | 6.1626739 |
| 259 | ZINC58975170 | 4.2000442 |
| 260 | ZINC21879601 | 5.676574 |
| 261 | ZINC31730871 | 5.8161331 |
| 262 | ZINC13139521 | 5.3939785 |
| 263 | ZINC27521214 | 6.4333844 |
| 264 | ZINC11527444 | 4.2741463 |
| 265 | ZINC09593675 | 5.7026528 |
| 266 | ZINC03336965 | 4.8918654 |
| 267 | ZINC02182850 | 5.1143864 |
| 268 | ZINC12565646 | 6.0696749 |
| 269 | ZINC09127339 | 4.8089448 |
| 270 | ZINC19237570 | 4.8518646 |
| 271 | ZINC09846789 | 4.629559 |
| 272 | ZINC16399460 | 5.3111675 |
| 273 | ZINC09871611 | 4.250365 |
| 274 | ZINC03282611 | 4.8454737 |
| 275 | ZINC10243604 | 5.2765793 |
| 276 | ZINC12768686 | 5.5750306 |
| 277 | ZINC63773311 | 6.0845196 |
| 278 | ZINC14383059 | 5.4626178 |
| 279 | ZINC28950583 | 4.8288186 |
| 280 | ZINC09540835 | 4.6606025 |
| 281 | ZINC22274916 | 4.4172264 |
| 282 | ZINC58168718 | 5.935826 |
| 283 | ZINC03269460 | 5.949101 |
| 284 | ZINC13014020 | 5.0615017 |
| 285 | ZINC01700878 | 4.6227087 |
| 286 | ZINC25231437 | 6.2427218 |
| 287 | ZINC89447974 | 6.1660636 |
| 288 | ZINC13465273 | 5.1111906 |
| 289 | ZINC12633361 | 5.4756431 |
| 290 | ZINC40174909 | 4.8098915 |
| 291 | ZINC13024174 | 5.6787896 |
| 292 | ZINC36245336 | 5.0754644 |
| 293 | ZINC10206589 | 5.3794716 |
| 294 | ZINC19572396 | 5.3381194 |
| 295 | ZINC64669563 | 5.0149207 |
| 296 | ZINC02301075 | 4.7553446 |
| 297 | ZINC17577419 | 5.0055743 |
| 298 | ZINC15730272 | 5.9474253 |
| 299 | ZINC06101367 | 5.2441459 |
| 300 | ZINC79720152 | 5.1077467 |
| 301 | ZINC16681334 | 4.4819636 |
| 302 | ZINC09028171 | 5.1936565 |
| 303 | ZINC13120257 | 4.1616536 |
| 304 | ZINC20628814 | 5.6735269 |
| 305 | ZINC33058604 | 5.4297598 |
| 306 | ZINC33039947 | 6.3576975 |
| 307 | ZINC06090157 | 5.452142 |
| 308 | ZINC89865656 | 4.842083 |
| 309 | ZINC03373950 | 4.2993892 |
| 310 | ZINC39446161 | 5.9476192 |
| 311 | ZINC28192577 | 4.2171486 |
| 312 | ZINC89835021 | 6.2580248 |
| 313 | ZINC02275866 | 3.6785453 |
| 314 | ZINC08382970 | 4.5781329 |
| 315 | ZINC85649767 | 4.723115 |
| 316 | ZINC45940756 | 5.1632465 |
| 317 | ZINC09106916 | 4.7559118 |
| 318 | ZINC65469626 | 6.3288261 |
| 319 | ZINC23380228 | 5.3589696 |
| 320 | ZINC08746829 | 4.228294 |
| 321 | ZINC02108974 | 6.8283088 |
| 322 | ZINC02341716 | 5.7376882 |
| 323 | ZINC05861863 | 5.8525131 |
| 324 | ZINC21655427 | 6.1948526 |
| 325 | ZINC14994333 | 6.398591 |
| 326 | ZINC13109739 | 5.05218 |
| 327 | ZINC59264758 | 4.7828759 |
| 328 | ZINC13450442 | 5.1853931 |
| 329 | ZINC35549572 | 5.7369374 |
| 330 | ZINC09235406 | 4.5757278 |
| 331 | ZINC12614534 | 4.5035112 |
| 332 | ZINC24875126 | 5.0732872 |
| 333 | ZINC36611590 | 5.5444038 |
| 334 | ZINC40782365 | 4.1339169 |
| 335 | ZINC00999608 | 4.8608946 |
| 336 | ZINC03338831 | 4.4012207 |
| 337 | ZINC12401765 | 4.0894689 |
| 338 | ZINC07778288 | 6.1617864 |
| 339 | ZINC64775492 | 6.5959729 |
| 340 | ZINC56643886 | 5.2859106 |
| 341 | ZINC67427385 | 5.5820559 |
| 342 | ZINC94539578 | 5.2244096 |
| 343 | ZINC35975348 | 5.1479992 |
| 344 | ZINC09250947 | 4.5091441 |
| 345 | ZINC64362156 | 5.1229929 |
| 346 | ZINC03301579 | 4.8256831 |
| 347 | ZINC83937507 | 5.7344355 |
| 348 | ZINC09518207 | 5.4608096 |
| 349 | ZINC18181258 | 5.0042914 |
| 350 | ZINC35314346 | 4.3570644 |
| 351 | ZINC06146094 | 6.077926 |
| 352 | ZINC09061690 | 5.177988 |
| 353 | ZINC00633236 | 4.7464243 |
| 354 | ZINC02388285 | 4.3416425 |
| 355 | ZINC13042382 | 5.3154256 |
| 356 | ZINC13496922 | 5.3613812 |
| 357 | ZINC03292885 | 6.1955251 |
| 358 | ZINC71965207 | 5.1139917 |
| 359 | ZINC14952759 | 5.6161221 |
| 360 | ZINC14273442 | 6.0572114 |
| 361 | ZINC40050866 | 5.3552283 |
| 362 | ZINC03848525 | 4.509807 |
| 363 | ZINC15729213 | 6.0602393 |
| 364 | ZINC19853567 | 5.3969517 |
| 365 | ZINC12924001 | 5.8894006 |
| 366 | ZINC06395255 | 5.1652139 |
| 367 | ZINC03840904 | 5.6804558 |
| 368 | ZINC12472821 | 7.3142547 |
| 369 | ZINC03268886 | 5.4581846 |
| 370 | ZINC40169295 | 5.5255496 |
| 371 | ZINC40142385 | 4.951594 |
| 372 | ZINC70697743 | 4.6606736 |
| 373 | ZINC89324781 | 6.3819226 |
| 374 | ZINC78816186 | 4.2014322 |
| 375 | ZINC09518744 | 5.7131165 |
| 376 | ZINC48103068 | 5.3528216 |
| 377 | ZINC05198461 | 5.4391216 |
| 378 | ZINC03018526 | 5.1539314 |
| 379 | ZINC33040481 | 5.2047613 |
| 380 | ZINC32971943 | 5.1149108 |
| 381 | ZINC55160132 | 5.3472954 |
| 382 | ZINC89312816 | 5.9371148 |
| 383 | ZINC36559260 | 5.9663094 |
| 384 | ZINC16737098 | 4.4861636 |
| 385 | ZINC03510134 | 4.0584595 |
| 386 | ZINC02092964 | 5.242854 |
| 387 | ZINC21882419 | 5.4136633 |
| 388 | ZINC16682562 | 5.6000322 |
| 389 | ZINC10220686 | 5.6202573 |
| 390 | ZINC45925115 | 5.0770644 |
| 391 | ZINC05348874 | 5.2010069 |
| 392 | ZINC38555855 | 6.0779706 |
| 393 | ZINC92778071 | 5.0644149 |
| 394 | ZINC16322421 | 6.4827851 |
| 395 | ZINC00877433 | 5.3485222 |
| 396 | ZINC33020839 | 5.5481187 |
| 397 | ZINC32929448 | 4.5118812 |
| 398 | ZINC02956023 | 5.2677287 |
| 399 | ZINC30985781 | 5.1827416 |
| 400 | ZINC13021965 | 6.1127215 |
| 401 | ZINC58179486 | 4.0721716 |
| 402 | ZINC40169978 | 5.5354491 |
| 403 | ZINC32686472 | 4.9764201 |
| 404 | ZINC79181992 | 5.4168604 |
| 405 | ZINC06039949 | 5.1367487 |
| 406 | ZINC22409680 | 6.5752791 |
| 407 | ZINC71589538 | 6.3380285 |
| 408 | ZINC09782877 | 5.7368774 |
| 409 | ZINC92231662 | 4.7132598 |
| 410 | ZINC12206261 | 6.4554684 |
| 411 | ZINC02306506 | 5.4291211 |
| 412 | ZINC38719895 | 6.019508 |
| 413 | ZINC72009309 | 5.6626287 |
| 414 | ZINC38741334 | 5.2388892 |
| 415 | ZINC09152139 | 5.2839772 |
| 416 | ZINC84338580 | 5.5136583 |
| 417 | ZINC03287706 | 4.7243186 |
| 418 | ZINC19938414 | 4.3675378 |
| 419 | ZINC21371533 | 5.0361794 |
| 420 | ZINC15466350 | 5.7218642 |
| 421 | ZINC14800213 | 6.9095584 |
| 422 | ZINC19320888 | 4.5604586 |
| 423 | ZINC15944122 | 6.1467242 |
| 424 | ZINC03106257 | 4.9244779 |
| 425 | ZINC20115768 | 6.6944716 |
| 426 | ZINC06910912 | 4.8458273 |
| 427 | ZINC15676155 | 5.5504187 |
| 428 | ZINC79484350 | 5.1228992 |
| 429 | ZINC04866558 | 4.5319385 |
| 430 | ZINC27526201 | 4.387389 |
| 431 | ZINC59487757 | 5.0899018 |
| 432 | ZINC46145722 | 5.0142682 |
| 433 | ZINC39845603 | 5.8779405 |
| 434 | ZINC03309252 | 6.0434342 |
| 435 | ZINC59489700 | 4.0387902 |
| 436 | ZINC83570572 | 6.4764422 |
| 437 | ZINC06379404 | 4.6072152 |
| 438 | ZINC09044958 | 5.7477037 |
| 439 | ZINC95363967 | 6.6766712 |
| 440 | ZINC02659694 | 6.552632 |
| 441 | ZINC61720005 | 4.8200175 |
| 442 | ZINC08662737 | 5.8098623 |
| 443 | ZINC59474330 | 4.5149113 |
| 444 | ZINC76870423 | 5.314252 |
| 445 | ZINC11235665 | 5.245236 |
| 446 | ZINC30994661 | 5.2571432 |
| 447 | ZINC93351127 | 5.1723452 |
| 448 | ZINC08484556 | 3.946446 |
| 449 | ZINC33271091 | 4.6135831 |
| 450 | ZINC49413175 | 5.9076655 |
| 451 | ZINC33078882 | 5.4844251 |
| 452 | ZINC12526489 | 5.4732238 |
| 453 | ZINC12935363 | 5.6116347 |
| 454 | ZINC09265831 | 6.0129453 |
| 455 | ZINC38534695 | 5.4052485 |
| 456 | ZINC89247215 | 4.5128983 |
| 457 | ZINC72248895 | 4.5916151 |
| 458 | ZINC12864958 | 4.4882358 |
| 459 | ZINC91657368 | 4.9991359 |
| 460 | ZINC02207433 | 4.9239548 |
| 461 | ZINC87320438 | 4.9256416 |
| 462 | ZINC08749012 | 4.7412571 |
| 463 | ZINC10296868 | 4.8095689 |
| 464 | ZINC01375690 | 4.8376285 |
| 465 | ZINC03401795 | 5.7473629 |
| 466 | ZINC02338454 | 4.4797179 |
| 467 | ZINC51364561 | 6.2503845 |
| 468 | ZINC33126210 | 4.6616187 |
| 469 | ZINC18194950 | 3.7018756 |
| 470 | ZINC20573445 | 5.8772795 |
| 471 | ZINC33414090 | 3.4607068 |
| 472 | ZINC86025409 | 5.0739513 |
| 473 | ZINC20607055 | 4.5565293 |
| 474 | ZINC13348223 | 5.0611225 |
| 475 | ZINC10944444 | 4.830082 |
| 476 | ZINC32808734 | 6.6944955 |
| 477 | ZINC03242439 | 4.2497192 |
| 478 | ZINC09559553 | 6.1944498 |
| 479 | ZINC03256688 | 7.7464356 |
| 480 | ZINC06498465 | 5.6192491 |
| 481 | ZINC10913405 | 5.222843 |
| 482 | ZINC16952310 | 5.0042691 |
| 483 | ZINC21604398 | 5.4395158 |
| 484 | ZINC01782246 | 4.3186466 |
| 485 | ZINC08425946 | 5.1945214 |
| 486 | ZINC40755353 | 4.3349358 |
| 487 | ZINC12702541 | 6.0628673 |
| 488 | ZINC65609364 | 4.6215359 |
| 489 | ZINC21886809 | 4.4508772 |
| 490 | ZINC92315049 | 5.9350351 |
| 491 | ZINC78873926 | 5.7950871 |
| 492 | ZINC33068302 | 7.523966 |
| 493 | ZINC78550185 | 4.3601111 |
| 494 | ZINC21664388 | 5.1745502 |
| 495 | ZINC69896444 | 4.3132089 |
| 496 | ZINC03412379 | 4.5948131 |
| 497 | ZINC12368218 | 6.8903546 |
| 498 | ZINC45891002 | 4.636792 |
| 499 | ZINC03244372 | 4.8328993 |
| 500 | ZINC11241817 | 6.1322771 |
| 501 | ZINC38519865 | 5.6019273 |
| 502 | ZINC20616835 | 3.8214401 |
| 503 | ZINC22629102 | 4.9350555 |
| 504 | ZINC01907048 | 5.920683 |
| 505 | ZINC23235314 | 4.6329653 |
| 506 | ZINC25782739 | 6.1345327 |
| 507 | ZINC13362304 | 5.0314972 |
| 508 | ZINC88630862 | 4.2979388 |
| 509 | ZINC33369170 | 5.3721861 |
| 510 | ZINC71981994 | 5.0648355 |
| 511 | ZINC03348533 | 6.820909 |
| 512 | ZINC67805165 | 5.685737 |
| 513 | ZINC05702384 | 3.6182444 |
| 514 | ZINC09442072 | 4.7455453 |
| 515 | ZINC09517962 | 4.9910183 |
| 516 | ZINC03323077 | 5.4042613 |
| 517 | ZINC09455844 | 5.2032072 |
| 518 | ZINC12053264 | 6.1826657 |
| 519 | ZINC90070481 | 4.7030548 |
| 520 | ZINC08426967 | 3.9869374 |
| 521 | ZINC11935370 | 4.9451399 |
| 522 | ZINC12483613 | 5.8760562 |
| 523 | ZINC12371448 | 6.5738276 |
| 524 | ZINC03302319 | 5.9188498 |
| 525 | ZINC21619358 | 5.5883807 |
| 526 | ZINC38509180 | 5.5170361 |
| 527 | ZINC41653283 | 4.0165122 |
| 528 | ZINC08726615 | 5.2337039 |
| 529 | ZINC03303989 | 6.0431835 |
| 530 | ZINC01238070 | 6.2046251 |
| 531 | ZINC67952476 | 4.6892861 |
| 532 | ZINC12932384 | 4.380111 |
| 533 | ZINC20838869 | 5.7173402 |
| 534 | ZINC07118471 | 7.2313534 |
| 535 | ZINC40192559 | 4.6669041 |
| 536 | ZINC65472917 | 3.9646277 |
| 537 | ZINC12757384 | 5.9659612 |
| 538 | ZINC65000455 | 6.5637076 |
| 539 | ZINC20861072 | 5.367407 |
| 540 | ZINC69950998 | 6.3411317 |
| 541 | ZINC72305596 | 5.0361204 |
| 542 | ZINC79924860 | 4.9762257 |
| 543 | ZINC62582465 | 4.6033227 |
| 544 | ZINC21821094 | 5.3685824 |
| 545 | ZINC04119527 | 3.6871717 |
| 546 | ZINC43103087 | 4.5736071 |
| 547 | ZINC67320182 | 5.9990585 |
| 548 | ZINC08739147 | 5.9013221 |
| 549 | ZINC64590618 | 5.9478974 |
| 550 | ZINC09254438 | 5.6082845 |
| 551 | ZINC28235022 | 5.9801612 |
| 552 | ZINC24908297 | 6.7264977 |
| 553 | ZINC73752476 | 4.414654 |
| 554 | ZINC84452354 | 4.9874825 |
| 555 | ZINC14553707 | 5.7223267 |
| 556 | ZINC72476226 | 6.5117477 |
| 557 | ZINC90644574 | 5.6084927 |
| 558 | ZINC09349262 | 4.6465297 |
| 559 | ZINC77917273 | 4.3696642 |
| 560 | ZINC95062548 | 4.4177743 |
| 561 | ZINC88235128 | 4.8079265 |
| 562 | ZINC40151552 | 3.6126332 |
| 563 | ZINC95073766 | 5.7146329 |
| 564 | ZINC90507441 | 4.5686727 |
| 565 | ZINC01572300 | 6.1723765 |
| 566 | ZINC26259559 | 4.6683657 |
| 567 | ZINC91535950 | 5.7121129 |
| 568 | ZINC95462400 | 4.9066745 |
| 569 | ZINC10877589 | 5.3883721 |
| 570 | ZINC01812476 | 4.661478 |
| 571 | ZINC40707884 | 4.819376 |
| 572 | ZINC09152065 | 5.1571455 |
| 573 | ZINC41644901 | 4.966222 |
| 574 | ZINC12983983 | 4.8041008 |
| 575 | ZINC78010846 | 5.050135 |
| 576 | ZINC31817786 | 4.4229518 |
| 577 | ZINC49472086 | 5.5019704 |
| 578 | ZINC08921278 | 5.3900378 |
| 579 | ZINC04612962 | 4.2763329 |
| 580 | ZINC32950556 | 5.1363085 |
| 581 | ZINC22579697 | 5.4675746 |
| 582 | ZINC17304241 | 4.2250508 |
| 583 | ZINC13016803 | 5.2356159 |
| 584 | ZINC09569137 | 6.526488 |
| 585 | ZINC21738183 | 4.7098164 |
| 586 | ZINC20375493 | 4.5385612 |
| 587 | ZINC06651442 | 4.8468212 |
| 588 | ZINC33113547 | 4.2606588 |
| 589 | ZINC09407510 | 5.5260784 |
| 590 | ZINC09394687 | 6.2511293 |
| 591 | ZINC72405867 | 5.6416673 |
| 592 | ZINC14139150 | 5.0027375 |
| 593 | ZINC77753025 | 4.8939366 |
| 594 | ZINC20248321 | 4.9127374 |
| 595 | ZINC12630081 | 5.1002527 |
| 596 | ZINC40151555 | 5.0838215 |
| 597 | ZINC35311257 | 4.9905713 |
| 598 | ZINC21177855 | 5.0247877 |
| 599 | ZINC49504958 | 5.8261311 |
| 600 | ZINC16956921 | 4.0681576 |
| 601 | ZINC08824932 | 6.3628788 |
| 602 | ZINC09042373 | 4.8836228 |
| 603 | ZINC16176149 | 5.6374085 |
| 604 | ZINC10641561 | 4.9239147 |
| 605 | ZINC92591920 | 5.0497654 |
| 606 | ZINC00572747 | 5.7201834 |
| 607 | ZINC16753982 | 5.6964283 |
| 608 | ZINC14086197 | 5.0020655 |
| 609 | ZINC00086154 | 5.2482598 |
| 610 | ZINC09512333 | 6.2598096 |
| 611 | ZINC72465635 | 5.2236005 |
| 612 | ZINC11937332 | 5.6031825 |
| 613 | ZINC89623966 | 4.7819105 |
| 614 | ZINC09152835 | 5.5968817 |
| 615 | ZINC91187081 | 5.1541995 |
| 616 | ZINC03317854 | 4.6513353 |
| 617 | ZINC20459036 | 6.2225074 |
| 618 | ZINC02079559 | 5.7303133 |
| 619 | ZINC65752743 | 5.288403 |
| 620 | ZINC13682266 | 6.4485767 |
| 621 | ZINC59857013 | 5.5228735 |
| 622 | ZINC12523749 | 5.1530979 |
| 623 | ZINC59563980 | 5.278109 |
| 624 | ZINC09631009 | 5.4718647 |
| 625 | ZINC08074039 | 6.0337496 |
| 626 | ZINC47173567 | 6.527095 |
| 627 | ZINC03393312 | 3.5163297 |
| 628 | ZINC83576963 | 4.6960421 |
| 629 | ZINC93088895 | 5.4461692 |
| 630 | ZINC15590075 | 5.0739871 |
| 631 | ZINC72439603 | 3.9131117 |
| 632 | ZINC09926403 | 4.1474267 |
| 633 | ZINC30663003 | 5.5255573 |
| 634 | ZINC63763351 | 4.4140736 |
| 635 | ZINC12217345 | 4.8435647 |
| 636 | ZINC41667803 | 5.1354485 |
| 637 | ZINC09540011 | 5.226944 |
| 638 | ZINC47162305 | 4.5906708 |
| 639 | ZINC07956035 | 4.1759155 |
| 640 | ZINC09244925 | 5.8786212 |
| 641 | ZINC08613118 | 5.0670286 |
| 642 | ZINC93260142 | 5.1586764 |
| 643 | ZINC12607300 | 4.9932356 |
| 644 | ZINC49451235 | 5.6802967 |
| 645 | ZINC42199386 | 5.4080971 |
| 646 | ZINC34813129 | 4.9574904 |
| 647 | ZINC03128201 | 4.8693253 |
| 648 | ZINC41527935 | 4.359943 |
| 649 | ZINC00949134 | 6.054819 |
| 650 | ZINC02206634 | 4.3444472 |
| 651 | ZINC44227605 | 5.2533401 |
| 652 | ZINC06152412 | 6.5874704 |
| 653 | ZINC25763682 | 4.4538145 |
| 654 | ZINC40169630 | 4.950554 |
| 655 | ZINC05896439 | 4.8047443 |
| 656 | ZINC80363018 | 6.1612387 |
| 657 | ZINC22636585 | 4.8724009 |
| 658 | ZINC20343157 | 5.448224 |
| 659 | ZINC92856203 | 4.7369275 |
| 660 | ZINC19881238 | 5.1829082 |
| 661 | ZINC92061549 | 4.7523377 |
| 662 | ZINC19346150 | 5.6995832 |
| 663 | ZINC16843739 | 5.2627137 |
| 664 | ZINC09472525 | 4.8836647 |
| 665 | ZINC10400433 | 6.6797611 |
| 666 | ZINC59922199 | 6.8124921 |
| 667 | ZINC78873283 | 5.9764684 |
| 668 | ZINC84075434 | 4.9363523 |
| 669 | ZINC78535734 | 4.0162743 |
| 670 | ZINC92271772 | 4.7981723 |
| 671 | ZINC91184248 | 4.3770404 |
| 672 | ZINC01318783 | 3.1693646 |
| 673 | ZINC09290111 | 3.7734085 |
| 674 | ZINC16608652 | 5.6864707 |
| 675 | ZINC08427869 | 5.1521337 |
| 676 | ZINC09739654 | 4.5300632 |
| 677 | ZINC04529947 | 3.3927585 |
| 678 | ZINC03298806 | 4.7972534 |
| 679 | ZINC79048931 | 5.114294 |
| 680 | ZINC89569616 | 6.0928024 |
| 681 | ZINC32573505 | 6.3152557 |
| 682 | ZINC76905024 | 5.4333509 |
| 683 | ZINC04945425 | 3.9200992 |
| 684 | ZINC12676532 | 4.4133912 |
| 685 | ZINC19337689 | 4.2761732 |
| 686 | ZINC69373331 | 4.9975161 |
| 687 | ZINC72321784 | 5.2060011 |
| 688 | ZINC81463630 | 4.9348724 |
| 689 | ZINC10157062 | 4.7114674 |
| 690 | ZINC02798582 | 5.6168297 |
| 691 | ZINC35313042 | 5.5209846 |
| 692 | ZINC12432570 | 6.5048911 |
| 693 | ZINC87423370 | 5.097359 |
| 694 | ZINC75286064 | 3.9987788 |
| 695 | ZINC12539900 | 4.8214463 |
| 696 | ZINC33140655 | 4.6928887 |
| 697 | ZINC49448477 | 5.4086226 |
| 698 | ZINC93682714 | 5.5036767 |
| 699 | ZINC01241829 | 4.1431828 |
| 700 | ZINC73680402 | 4.617669 |
| 701 | ZINC73740468 | 5.0708885 |
| 702 | ZINC12242628 | 6.6568653 |
| 703 | ZINC84556177 | 5.9222699 |
| 704 | ZINC73038135 | 4.09449 |
| 705 | ZINC02434639 | 4.947971 |
| 706 | ZINC72338737 | 5.7584045 |
| 707 | ZINC91897316 | 4.598344 |
| 708 | ZINC12793757 | 4.8037946 |
| 709 | ZINC09269772 | 5.262708 |
| 710 | ZINC89780576 | 4.967667 |
| 711 | ZINC69292456 | 5.8372961 |
| 712 | ZINC68668081 | 4.5709092 |
| 713 | ZINC39388732 | 3.0263956 |
| 714 | ZINC93089242 | 4.2431961 |
| 715 | ZINC01085381 | 5.1075538 |
| 716 | ZINC72134753 | 4.8943495 |
| 717 | ZINC78952910 | 4.2705435 |
| 718 | ZINC12063808 | 6.4578751 |
| 719 | ZINC39916806 | 6.5511217 |
| 720 | ZINC20738908 | 4.0714363 |
| 721 | ZINC33513637 | 4.6092232 |
| 722 | ZINC10641141 | 4.7698503 |
| 723 | ZINC78404714 | 5.7795467 |
| 724 | ZINC54672266 | 5.6932613 |
| 725 | ZINC24838920 | 5.1498602 |
| 726 | ZINC92119657 | 4.0942411 |
| 727 | ZINC04200986 | 5.187087 |
| 728 | ZINC72443763 | 5.7637815 |
| 729 | ZINC84876246 | 3.8460745 |
| 730 | ZINC15358161 | 4.3491484 |
| 731 | ZINC95450700 | 5.0735396 |
| 732 | ZINC13361151 | 5.302646 |
| 733 | ZINC19297424 | 5.7126773 |
| 734 | ZINC09065330 | 4.7631202 |
| 735 | ZINC55765566 | 5.0264688 |
| 736 | ZINC20414315 | 4.7019701 |
| 737 | ZINC64102052 | 6.468087 |
| 738 | ZINC47638994 | 5.0680913 |
| 739 | ZINC06602344 | 5.6610135 |
| 740 | ZINC76961114 | 5.2492083 |
| 741 | ZINC71930576 | 4.8894813 |
| 742 | ZINC73378198 | 5.7203278 |
| 743 | ZINC19742140 | 6.3542791 |
| 744 | ZINC46086684 | 4.671075 |
| 745 | ZINC93089253 | 4.9580299 |
| 746 | ZINC06597096 | 4.3321548 |
| 747 | ZINC81704672 | 6.1526887 |
| 748 | ZINC72880195 | 4.5051297 |
| 749 | ZINC92874180 | 5.4017947 |
| 750 | ZINC08831860 | 4.8389639 |
| 751 | ZINC15345167 | 4.8771511 |
| 752 | ZINC90222811 | 4.9302215 |
| 753 | ZINC92788359 | 5.0287563 |
| 754 | ZINC08956068 | 6.2098134 |
| 755 | ZINC90503796 | 4.9698441 |
| 756 | ZINC16844061 | 4.5821898 |
| 757 | ZINC78618795 | 5.3841415 |
| 758 | ZINC04423366 | 6.0107687 |
| 759 | ZINC13525575 | 4.5504085 |
| 760 | ZINC07777384 | 4.5950692 |
| 761 | ZINC11452422 | 6.5494958 |
| 762 | ZINC14016665 | 5.2503012 |
| 763 | ZINC57790935 | 4.7933122 |
| 764 | ZINC03294973 | 5.176256 |
| 765 | ZINC44479691 | 5.0594933 |
| 766 | ZINC57830415 | 4.5847037 |
| 767 | ZINC72444911 | 5.3078191 |
| 768 | ZINC10276391 | 4.4470085 |
| 769 | ZINC12447923 | 5.0740628 |
| 770 | ZINC71946891 | 5.8321953 |
| 771 | ZINC16683385 | 4.0885641 |
| 772 | ZINC40178340 | 5.7147378 |
| 773 | ZINC03014309 | 4.4946248 |
| 774 | ZINC92152701 | 4.8547977 |
| 775 | ZINC04908203 | 5.7961902 |
| 776 | ZINC01143356 | 4.0796167 |
| 777 | ZINC90706354 | 4.7967492 |
| 778 | ZINC09986133 | 5.1290293 |
| 779 | ZINC01245316 | 5.2461354 |
| 780 | ZINC15772715 | 4.0324326 |
| 781 | ZINC77676912 | 4.621773 |
| 782 | ZINC71766836 | 5.4278707 |
| 783 | ZINC77983603 | 5.3009672 |
| 784 | ZINC47200574 | 5.3391152 |
| 785 | ZINC01112751 | 6.2124282 |
| 786 | ZINC73873271 | 5.1485118 |
| 787 | ZINC02430278 | 5.2191336 |
| 788 | ZINC12484047 | 4.726169 |
| 789 | ZINC09334921 | 4.4887787 |
| 790 | ZINC44968040 | 4.9421267 |
| 791 | ZINC02079670 | 4.0227854 |
| 792 | ZINC20494888 | 5.2482843 |
| 793 | ZINC20354470 | 4.3753347 |
| 794 | ZINC44166647 | 5.0420186 |
| 795 | ZINC04373695 | 6.0645681 |
| 796 | ZINC02663936 | 5.8349219 |
| 797 | ZINC79983075 | 5.1381135 |
| 798 | ZINC01094949 | 4.6046172 |
| 799 | ZINC09645143 | 4.4742251 |
| 800 | ZINC09996221 | 5.1252979 |
| 801 | ZINC13360898 | 4.6481324 |
| 802 | ZINC36676484 | 6.3184305 |
| 803 | ZINC77955411 | 4.3438345 |
| 804 | ZINC09289263 | 5.5394101 |
| 805 | ZINC25063926 | 3.8200548 |
| 806 | ZINC00201488 | 3.8517075 |
| 807 | ZINC09765687 | 5.7752564 |
| 808 | ZINC29872278 | 5.849885 |
| 809 | ZINC09052314 | 4.8127859 |
| 810 | ZINC33291802 | 5.9303236 |
| 811 | ZINC12241866 | 6.96525 |
| 812 | ZINC67475598 | 6.4974618 |
| 813 | ZINC79000789 | 5.6968596 |
| 814 | ZINC92780854 | 4.9973517 |
| 815 | ZINC36154785 | 4.4655571 |
| 816 | ZINC02748315 | 4.6113722 |
| 817 | ZINC80362617 | 4.497438 |
| 818 | ZINC21370941 | 4.3711601 |
| 819 | ZINC08655349 | 4.7101341 |
| 820 | ZINC09368595 | 5.484471 |
| 821 | ZINC92389008 | 4.9752296 |
| 822 | ZINC92346763 | 4.7157799 |
| 823 | ZINC08994660 | 5.3045479 |
| 824 | ZINC04221767 | 5.5067097 |
| 825 | ZINC02060033 | 5.2678571 |
| 826 | ZINC91056304 | 5.6975505 |
| 827 | ZINC19897690 | 4.8196945 |
| 828 | ZINC33411746 | 5.9557713 |
| 829 | ZINC92023681 | 4.8808054 |
| 830 | ZINC15674639 | 4.7665177 |
| 831 | ZINC91590990 | 5.6053597 |
| 832 | ZINC21537199 | 5.2251108 |
| 833 | ZINC09926407 | 6.1233752 |
| 834 | ZINC09369061 | 4.5518312 |
| 835 | ZINC08931895 | 5.6719869 |
| 836 | ZINC09711888 | 5.1056252 |
| 837 | ZINC33283914 | 5.6251195 |
| 838 | ZINC15776391 | 4.6775948 |
| 839 | ZINC56925839 | 5.9051164 |
| 840 | ZINC72581141 | 5.1472738 |
| 841 | ZINC01047103 | 5.5632378 |
| 842 | ZINC13635634 | 4.4799751 |
| 843 | ZINC11726471 | 5.8579333 |
| 844 | ZINC72013850 | 5.809086 |
| 845 | ZINC65304903 | 4.9817771 |
| 846 | ZINC01425752 | 4.614509 |
| 847 | ZINC72404146 | 6.1327397 |
| 848 | ZINC09753540 | 6.1794897 |
| 849 | ZINC81766378 | 5.3934706 |
| 850 | ZINC58422275 | 4.8170934 |
| 851 | ZINC73051858 | 4.7346696 |
| 852 | ZINC22056188 | 4.7892326 |
| 853 | ZINC01044858 | 4.1756855 |
| 854 | ZINC41077443 | 4.9512175 |
| 855 | ZINC02086640 | 4.6366859 |
| 856 | ZINC92778467 | 5.1652749 |
| 857 | ZINC11991501 | 4.4227618 |
| 858 | ZINC05245843 | 5.8647647 |
| 859 | ZINC02800386 | 5.1994107 |
| 860 | ZINC32593675 | 5.381431 |
| 861 | ZINC86371128 | 4.1769925 |
| 862 | ZINC90227423 | 4.8604103 |
| 863 | ZINC91341794 | 5.5958972 |
| 864 | ZINC77926468 | 6.2201507 |
| 865 | ZINC46581368 | 4.0725326 |
| 866 | ZINC04200999 | 5.3656151 |
| 867 | ZINC03296199 | 6.1577636 |
| 868 | ZINC32977957 | 5.7229633 |
| 869 | ZINC22311009 | 4.8869283 |
| 870 | ZINC90088401 | 4.8714624 |
| 871 | ZINC12796051 | 5.3444532 |
| 872 | ZINC38711180 | 4.1669353 |
| 873 | ZINC86830232 | 4.5773995 |
| 874 | ZINC67934935 | 6.1770345 |
| 875 | ZINC78764317 | 6.4014474 |
| 876 | ZINC36392899 | 4.0565125 |
| 877 | ZINC06076933 | 5.978853 |
| 878 | ZINC91301792 | 5.8563522 |
| 879 | ZINC66076687 | 3.6313215 |
| 880 | ZINC13720079 | 5.0355533 |
| 881 | ZINC79062434 | 4.1909712 |
| 882 | ZINC71990163 | 4.927832 |
| 883 | ZINC20332384 | 4.8726392 |
| 884 | ZINC02873082 | 4.6705464 |
| 885 | ZINC08448491 | 5.3804488 |
| 886 | ZINC69452058 | 4.6752545 |
| 887 | ZINC22760424 | 6.0327621 |
| 888 | ZINC59342127 | 3.9855774 |
| 889 | ZINC66935713 | 4.9194929 |
| 890 | ZINC49463710 | 5.9029676 |
| 891 | ZINC05083267 | 5.0459118 |
| 892 | ZINC53364891 | 5.1336311 |
| 893 | ZINC41394136 | 4.1596994 |
| 894 | ZINC77993357 | 5.4287019 |
| 895 | ZINC07225624 | 4.530349 |
| 896 | ZINC14250524 | 5.2021008 |
| 897 | ZINC59264491 | 4.7305053 |
| 898 | ZINC16545537 | 3.9991826 |
| 899 | ZINC86553589 | 4.7362965 |
| 900 | ZINC03412530 | 4.4916563 |
| 901 | ZINC95463060 | 5.7618263 |
| 902 | ZINC15863943 | 5.0692511 |
| 903 | ZINC11035560 | 5.1550785 |
| 904 | ZINC12471581 | 5.0473522 |
| 905 | ZINC91512471 | 4.8498264 |
| 906 | ZINC79052342 | 3.8868422 |
| 907 | ZINC89339903 | 4.3373903 |
| 908 | ZINC21892606 | 5.4720043 |
| 909 | ZINC56937062 | 3.6852217 |
| 910 | ZINC81229209 | 4.1485236 |
| 911 | ZINC91542181 | 5.0016185 |
| 912 | ZINC03274706 | 4.38649 |
| 913 | ZINC11650866 | 5.1140224 |
| 914 | ZINC33052014 | 5.858303 |
| 915 | ZINC71938681 | 5.8549258 |
| 916 | ZINC14649181 | 6.1559799 |
| 917 | ZINC39191084 | 5.3539057 |
| 918 | ZINC70868722 | 5.2191712 |
| 919 | ZINC03308990 | 5.4204384 |
| 920 | ZINC39231998 | 4.7172097 |
| 921 | ZINC09462128 | 5.0142218 |
| 922 | ZINC61402136 | 7.8335411 |
| 923 | ZINC21405033 | 5.0401641 |
| 924 | ZINC06677985 | 3.7096986 |
| 925 | ZINC03033350 | 4.9548588 |
| 926 | ZINC78237942 | 5.909646 |
| 927 | ZINC23144578 | 6.97739 |
| 928 | ZINC94671235 | 5.4315619 |
| 929 | ZINC14417739 | 5.5260704 |
| 930 | ZINC02073140 | 4.2939002 |
| 931 | ZINC75159903 | 5.7377577 |
| 932 | ZINC09496194 | 4.4575661 |
| 933 | ZINC12793386 | 4.3161551 |
| 934 | ZINC09594567 | 4.5063958 |
| 935 | ZINC00405760 | 5.3646155 |
| 936 | ZINC39955366 | 3.8301743 |
| 937 | ZINC65010201 | 5.5019764 |
| 938 | ZINC18325687 | 3.6909936 |
| 939 | ZINC78609153 | 5.791689 |
| 940 | ZINC15181537 | 4.7890445 |
| 941 | ZINC09461915 | 4.6011482 |
| 942 | ZINC62564065 | 5.1421392 |
| 943 | ZINC58124569 | 5.3333702 |
| 944 | ZINC40177922 | 5.3601836 |
| 945 | ZINC91941152 | 4.3330521 |
| 946 | ZINC00611340 | 4.8970461 |
| 947 | ZINC00933163 | 5.4378988 |
| 948 | ZINC77936642 | 6.7765689 |
| 949 | ZINC64875650 | 6.2791924 |
| 950 | ZINC38718102 | 4.4923605 |
| 951 | ZINC67946527 | 4.4933545 |
| 952 | ZINC72128396 | 5.2257441 |
| 953 | ZINC06177378 | 5.0059984 |
| 954 | ZINC34095348 | 7.25609 |
| 955 | ZINC71983476 | 5.6237086 |
| 956 | ZINC32852771 | 3.9814088 |
| 957 | ZINC93705771 | 4.8375745 |
| 958 | ZINC85393150 | 4.8771332 |
| 959 | ZINC24747008 | 4.3417292 |
| 960 | ZINC12775187 | 5.3601128 |
| 961 | ZINC27663358 | 5.0648296 |
| 962 | ZINC48290533 | 5.0369854 |
| 963 | ZINC09465529 | 4.9260546 |
| 964 | ZINC03335666 | 5.514324 |
| 965 | ZINC59263188 | 4.7668892 |
| 966 | ZINC79052787 | 4.9367161 |
| 967 | ZINC35313603 | 5.5977214 |
| 968 | ZINC09641665 | 4.8250654 |
| 969 | ZINC09283416 | 4.9006115 |
| 970 | ZINC10738650 | 5.6137706 |
| 971 | ZINC03048001 | 6.0762932 |
| 972 | ZINC09596057 | 4.2585589 |
| 973 | ZINC40211073 | 4.7538679 |
| 974 | ZINC88577473 | 5.6606954 |
| 975 | ZINC02808836 | 5.4063781 |
| 976 | ZINC71982060 | 5.1136057 |
| 977 | ZINC12631385 | 5.2934343 |
| 978 | ZINC35622806 | 5.7878797 |
| 979 | ZINC33257054 | 3.7157136 |
| 980 | ZINC72028715 | 4.7503466 |
| 981 | ZINC13592613 | 5.2597597 |
| 982 | ZINC91255539 | 4.5953567 |
| 983 | ZINC22902408 | 5.1277563 |
| 984 | ZINC71961272 | 5.9659472 |
| 985 | ZINC12911834 | 4.3979019 |
| 986 | ZINC64969959 | 5.0661479 |
| 987 | ZINC15015462 | 5.4931512 |
| 988 | ZINC14162515 | 5.4528369 |
| 989 | ZINC36619160 | 5.4937363 |
| 990 | ZINC36711768 | 5.6196627 |
| 991 | ZINC54641444 | 4.8831285 |
| 992 | ZINC03390423 | 3.8719196 |
| 993 | ZINC71819268 | 4.4554432 |
| 994 | ZINC25175542 | 4.7820337 |
| 995 | ZINC90608017 | 4.2888819 |
| 996 | ZINC01157917 | 4.71044 |
| 997 | ZINC41585222 | 4.7732298 |
| 998 | ZINC72329047 | 6.6047185 |
| 999 | ZINC59391344 | 4.9602893 |
| 1000 | ZINC14375370 | 5.6581599 |
| 1001 | ZINC13566512 | 5.3073566 |
| 1002 | ZINC40321736 | 4.7244086 |
| 1003 | ZINC93207330 | 4.7796021 |
| 1004 | ZINC77200563 | 4.5697764 |
| 1005 | ZINC22605910 | 4.5276402 |
| 1006 | ZINC00029618 | 3.5645932 |
| 1007 | ZINC45949837 | 4.3265308 |
| 1008 | ZINC72156255 | 4.3455054 |
| 1009 | ZINC76764608 | 6.0653124 |
| 1010 | ZINC08598036 | 4.6857546 |
| 1011 | ZINC13642266 | 4.6102066 |
| 1012 | ZINC09549415 | 5.079678 |
| 1013 | ZINC95447985 | 5.8433493 |
| 1014 | ZINC13549803 | 7.356818 |
| 1015 | ZINC40755849 | 4.5067526 |
| 1016 | ZINC11650124 | 5.0008252 |
| 1017 | ZINC13637557 | 4.6768973 |
| 1018 | ZINC04383859 | 4.7384773 |
| 1019 | ZINC67898358 | 5.2891475 |
| 1020 | ZINC24983658 | 3.9758661 |
| 1021 | ZINC90140548 | 4.9849582 |
| 1022 | ZINC08191226 | 5.4365648 |
| 1023 | ZINC17185687 | 4.5934551 |
| 1024 | ZINC01668251 | 4.4174175 |
| 1025 | ZINC81095698 | 4.885541 |
| 1026 | ZINC06674219 | 5.0934686 |
| 1027 | ZINC05100321 | 4.5717712 |
| 1028 | ZINC91294542 | 4.5486089 |
| 1029 | ZINC91452949 | 5.9022903 |
| 1030 | ZINC08193352 | 5.0461176 |
| 1031 | ZINC36353495 | 4.0095256 |
| 1032 | ZINC28284631 | 4.5181406 |
| 1033 | ZINC95363702 | 3.880005 |
| 1034 | ZINC71916222 | 5.9605879 |
| 1035 | ZINC46914745 | 4.4540541 |
| 1036 | ZINC03361880 | 4.7722384 |
| 1037 | ZINC04977910 | 4.8319681 |
| 1038 | ZINC09788891 | 3.9673522 |
| 1039 | ZINC16695667 | 4.3190149 |
| 1040 | ZINC29839237 | 5.2190286 |
| 1041 | ZINC57520297 | 4.9066396 |
| 1042 | ZINC13358814 | 5.688213 |
| 1043 | ZINC12913796 | 4.9150071 |
| 1044 | ZINC15018282 | 4.2081333 |
| 1045 | ZINC79035611 | 4.9799885 |
| 1046 | ZINC25015401 | 5.7148313 |
| 1047 | ZINC71804036 | 4.3607612 |
| 1048 | ZINC40448167 | 4.3671786 |
| 1049 | ZINC68898808 | 5.3957985 |
| 1050 | ZINC91854153 | 5.2922177 |
| 1051 | ZINC06134929 | 5.7998651 |
| 1052 | ZINC03169962 | 4.6831058 |
| 1053 | ZINC17130634 | 4.4484414 |
| 1054 | ZINC38865362 | 6.7150841 |
| 1055 | ZINC94910335 | 4.8968562 |
| 1056 | ZINC12559183 | 5.6383514 |
| 1057 | ZINC68727742 | 5.0915162 |
| 1058 | ZINC02335926 | 4.8203656 |
| 1059 | ZINC03212604 | 4.0209637 |
| 1060 | ZINC31157015 | 5.4691054 |
| 1061 | ZINC13095279 | 5.0357612 |
| 1062 | ZINC72432747 | 4.4757145 |
| 1063 | ZINC21033382 | 3.5971452 |
| 1064 | ZINC00210261 | 4.7910797 |
| 1065 | ZINC69721187 | 5.0084729 |
| 1066 | ZINC71926540 | 6.0747094 |
| 1067 | ZINC16323368 | 4.5501637 |
| 1068 | ZINC34900022 | 5.4055498 |
| 1069 | ZINC32924356 | 5.0267355 |
| 1070 | ZINC40221616 | 6.3691821 |
| 1071 | ZINC20109935 | 5.9527791 |
| 1072 | ZINC15733550 | 5.3160267 |
| 1073 | ZINC43746700 | 6.0956779 |
| 1074 | ZINC17865090 | 6.0457687 |
| 1075 | ZINC14962561 | 4.809836 |
| 1076 | ZINC55244578 | 5.790351 |
| 1077 | ZINC13635648 | 3.7506085 |
| 1078 | ZINC16436648 | 5.0405964 |
| 1079 | ZINC40983179 | 4.3292941 |
| 1080 | ZINC35955784 | 5.4257482 |
| 1081 | ZINC04571359 | 4.9262434 |
| 1082 | ZINC64954892 | 5.8704184 |
| 1083 | ZINC03399648 | 3.5680443 |
| 1084 | ZINC56208041 | 4.5470513 |
| 1085 | ZINC26802729 | 4.2607748 |
| 1086 | ZINC09825197 | 5.5433265 |
| 1087 | ZINC49059648 | 6.0762911 |
| 1088 | ZINC36366173 | 5.122886 |
| 1089 | ZINC66607014 | 4.6142935 |
| 1090 | ZINC85224367 | 3.8685684 |
| 1091 | ZINC00626967 | 4.4475858 |
| 1092 | ZINC72094275 | 6.3115532 |
| 1093 | ZINC03103563 | 5.4627774 |
| 1094 | ZINC89796671 | 5.1440225 |
| 1095 | ZINC03839189 | 5.0142259 |
| 1096 | ZINC72306739 | 4.9831864 |
| 1097 | ZINC12088544 | 6.0119607 |
| 1098 | ZINC92700469 | 3.9012625 |
| 1099 | ZINC95050342 | 4.8617864 |
| 1100 | ZINC12631381 | 4.8416151 |
| 1101 | ZINC32806452 | 3.5385305 |
| 1102 | ZINC75118196 | 4.7103848 |
| 1103 | ZINC94598239 | 4.2837753 |
| 1104 | ZINC09713708 | 4.6622254 |
| 1105 | ZINC95007093 | 4.78803 |
| 1106 | ZINC90100364 | 6.3774454 |
| 1107 | ZINC40399191 | 4.5154827 |
| 1108 | ZINC68768042 | 5.4496689 |
| 1109 | ZINC92127627 | 6.2687096 |
| 1110 | ZINC65116343 | 5.7219818 |
| 1111 | ZINC74433229 | 5.0799084 |
| 1112 | ZINC90049071 | 4.6499977 |
| 1113 | ZINC93938692 | 5.7318133 |
| 1114 | ZINC89509500 | 6.1526024 |
| 1115 | ZINC73406552 | 3.2339918 |
| 1116 | ZINC88099900 | 4.9277951 |
| 1117 | ZINC92467811 | 5.0452582 |
| 1118 | ZINC93433432 | 5.8457298 |
| 1119 | ZINC19462110 | 5.1077249 |
| 1120 | ZINC40106654 | 4.7531179 |
| 1121 | ZINC38620617 | 5.3759495 |
| 1122 | ZINC19983534 | 4.3811368 |
| 1123 | ZINC63872069 | 5.4025574 |
| 1124 | ZINC44967516 | 4.6615225 |
| 1125 | ZINC23243363 | 5.2967503 |
| 1126 | ZINC83197851 | 5.3727933 |
| 1127 | ZINC92870051 | 4.2427169 |
| 1128 | ZINC09250849 | 5.3440916 |
| 1129 | ZINC91690947 | 5.1440673 |
| 1130 | ZINC05100854 | 3.8073766 |
| 1131 | ZINC79498495 | 5.0598171 |
| 1132 | ZINC79005445 | 4.39584 |
| 1133 | ZINC72902133 | 5.3858018 |
| 1134 | ZINC49384054 | 4.6228545 |
| 1135 | ZINC75475518 | 4.4050646 |
| 1136 | ZINC75109966 | 4.4594774 |
| 1137 | ZINC49393147 | 4.2102055 |
| 1138 | ZINC89955822 | 4.4263729 |
| 1139 | ZINC16952402 | 4.5856887 |
| 1140 | ZINC93772337 | 4.9758146 |
| 1141 | ZINC01040844 | 6.4824108 |
| 1142 | ZINC86069151 | 6.1226101 |
| 1143 | ZINC06785265 | 5.9035451 |
| 1144 | ZINC15729959 | 5.2484457 |
| 1145 | ZINC17480217 | 5.333834 |
| 1146 | ZINC57486861 | 4.9637073 |
| 1147 | ZINC39898623 | 5.0506584 |
| 1148 | ZINC94771092 | 4.5688688 |
| 1149 | ZINC49422454 | 5.0573875 |
| 1150 | ZINC94434936 | 4.7364246 |
| 1151 | ZINC39983341 | 5.2554188 |
| 1152 | ZINC89200841 | 3.7537469 |
| 1153 | ZINC08690270 | 5.9964295 |
| 1154 | ZINC71969909 | 5.3623633 |
| 1155 | ZINC32602721 | 4.4101735 |
| 1156 | ZINC72430881 | 5.1669696 |
| 1157 | ZINC89599633 | 5.1875379 |
| 1158 | ZINC22707407 | 6.4888918 |
| 1159 | ZINC94993415 | 4.1832731 |
| 1160 | ZINC53335072 | 6.2091738 |
| 1161 | ZINC20136298 | 5.1748602 |
| 1162 | ZINC92325549 | 5.4920727 |
| 1163 | ZINC20682369 | 6.1939497 |
| 1164 | ZINC65373943 | 5.9550631 |
| 1165 | ZINC57478260 | 5.4366051 |
| 1166 | ZINC81675504 | 5.5214742 |
| 1167 | ZINC07952720 | 5.0168428 |
| 1168 | ZINC03179293 | 5.225616 |
| 1169 | ZINC11027440 | 5.3935209 |
| 1170 | ZINC37460542 | 4.3049812 |
| 1171 | ZINC11529949 | 6.2278644 |
| 1172 | ZINC91750453 | 4.7972507 |
| 1173 | ZINC55502694 | 4.181207 |
| 1174 | ZINC92231519 | 4.0469353 |
| 1175 | ZINC73879557 | 4.2050235 |
| 1176 | ZINC91941091 | 4.9568797 |
| 1177 | ZINC05868823 | 4.7304908 |
| 1178 | ZINC02637972 | 5.280442 |
| 1179 | ZINC80786071 | 6.6668958 |
| 1180 | ZINC71282795 | 4.9366716 |
| 1181 | ZINC62590096 | 6.6292789 |
| 1182 | ZINC01126006 | 4.4553553 |
| 1183 | ZINC05217069 | 5.4722335 |
| 1184 | ZINC77143349 | 4.5464757 |
| 1185 | ZINC13946354 | 4.6420791 |
| 1186 | ZINC49574659 | 4.890511 |
| 1187 | ZINC17300989 | 4.3797866 |
| 1188 | ZINC01736721 | 5.2951736 |
| 1189 | ZINC04411294 | 3.9939145 |
| 1190 | ZINC38776680 | 4.8228708 |
| 1191 | ZINC00871697 | 5.7581446 |
| 1192 | ZINC04783706 | 4.9670144 |
| 1193 | ZINC72320623 | 5.0280357 |
| 1194 | ZINC14163290 | 5.711411 |
| 1195 | ZINC05554441 | 4.28189 |
| 1196 | ZINC32500729 | 5.9769846 |
| 1197 | ZINC92777277 | 4.3296778 |
| 1198 | ZINC16545561 | 4.5390114 |
| 1199 | ZINC67967652 | 4.8503605 |
| 1200 | ZINC48375997 | 5.1823662 |
| 1201 | ZINC58393471 | 4.2182941 |
| 1202 | ZINC73383809 | 6.0550797 |
| 1203 | ZINC58292741 | 4.5551671 |
| 1204 | ZINC72238711 | 4.75503 |
| 1205 | ZINC10810330 | 4.6688795 |
| 1206 | ZINC68458997 | 5.1566091 |
| 1207 | ZINC92472937 | 5.8492135 |
| 1208 | ZINC79047979 | 5.1366525 |
| 1209 | ZINC92437283 | 4.6024985 |
| 1210 | ZINC12300244 | 6.0909733 |
| 1211 | ZINC89299362 | 4.5445085 |
| 1212 | ZINC21630600 | 4.6568199 |
| 1213 | ZINC12550559 | 4.9874094 |
| 1214 | ZINC72418732 | 5.2784623 |
| 1215 | ZINC79502154 | 5.066952 |
| 1216 | ZINC77948091 | 6.5067205 |
| 1217 | ZINC67427267 | 4.8556025 |
| 1218 | ZINC70954563 | 5.0037414 |
| 1219 | ZINC72192051 | 4.4420187 |
| 1220 | ZINC88046649 | 3.6897825 |
| 1221 | ZINC41644403 | 5.4260218 |
| 1222 | ZINC05198108 | 4.4241515 |
| 1223 | ZINC08417579 | 4.8799896 |
| 1224 | ZINC94210570 | 5.5277162 |
| 1225 | ZINC64868745 | 4.9074495 |
| 1226 | ZINC35299431 | 4.8355521 |
| 1227 | ZINC45506733 | 4.6040144 |
| 1228 | ZINC60530326 | 4.1790061 |
| 1229 | ZINC21959747 | 5.5440403 |
| 1230 | ZINC12370740 | 4.3261276 |
| 1231 | ZINC53748690 | 4.642815 |
| 1232 | ZINC86085764 | 4.7326382 |
| 1233 | ZINC86593651 | 4.5272173 |
| 1234 | ZINC35321232 | 4.1419919 |
| 1235 | ZINC40723475 | 4.301776 |
| 1236 | ZINC31085523 | 5.5461728 |
| 1237 | ZINC85975166 | 3.789552 |
| 1238 | ZINC31283527 | 5.7109464 |
| 1239 | ZINC93778980 | 4.6165013 |
| 1240 | ZINC91125054 | 3.9540385 |
| 1241 | ZINC06016668 | 4.9211878 |
| 1242 | ZINC49030833 | 3.9657524 |
| 1243 | ZINC13960685 | 4.655242 |
| 1244 | ZINC09690392 | 5.1774785 |
| 1245 | ZINC06487653 | 4.2824623 |
| 1246 | ZINC22198228 | 5.3336595 |
| 1247 | ZINC89891115 | 4.2636561 |
| 1248 | ZINC34145231 | 5.697446 |
| 1249 | ZINC78494970 | 5.8444866 |
| 1250 | ZINC94236349 | 5.1459884 |
| 1251 | ZINC41548407 | 5.2564486 |
| 1252 | ZINC10109513 | 5.1470082 |
| 1253 | ZINC73438432 | 4.1564757 |
| 1254 | ZINC11651390 | 3.9520675 |
| 1255 | ZINC44431449 | 5.5237192 |
| 1256 | ZINC48367683 | 4.2078053 |
| 1257 | ZINC15774244 | 5.1341252 |
| 1258 | ZINC31099599 | 5.1139923 |
| 1259 | ZINC89701029 | 4.7160431 |
| 1260 | ZINC02904724 | 4.8504285 |
| 1261 | ZINC08604659 | 5.0091976 |
| 1262 | ZINC38502783 | 4.3761355 |
| 1263 | ZINC76433082 | 4.1863583 |
| 1264 | ZINC12911166 | 4.1290102 |
| 1265 | ZINC84735476 | 4.8549411 |
| 1266 | ZINC94058317 | 5.6541852 |
| 1267 | ZINC93261144 | 5.5519455 |
| 1268 | ZINC41496608 | 5.3658829 |
| 1269 | ZINC40840767 | 6.2978675 |
| 1270 | ZINC91351100 | 5.9413531 |
| 1271 | ZINC05604464 | 3.4421379 |
| 1272 | ZINC41154792 | 5.3349078 |
| 1273 | ZINC87116914 | 5.1384803 |
| 1274 | ZINC80298035 | 5.8739658 |
| 1275 | ZINC25784032 | 4.1465535 |
| 1276 | ZINC09601013 | 5.5578083 |
| 1277 | ZINC81815470 | 4.9807838 |
| 1278 | ZINC89289828 | 4.8646524 |
| 1279 | ZINC89351867 | 4.8208426 |
| 1280 | ZINC71938994 | 5.3352411 |
| 1281 | ZINC12440961 | 6.665947 |
| 1282 | ZINC72226870 | 4.234933 |
| 1283 | ZINC91871903 | 4.9633333 |
| 1284 | ZINC05697037 | 4.1991801 |
| 1285 | ZINC79036542 | 5.4227659 |
| 1286 | ZINC95041275 | 4.8788711 |
| 1287 | ZINC52489037 | 4.6521363 |
| 1288 | ZINC38754338 | 5.1953034 |
| 1289 | ZINC94623702 | 4.4935723 |
| 1290 | ZINC07328247 | 5.0485204 |
| 1291 | ZINC33127666 | 5.6522909 |
| 1292 | ZINC69619777 | 4.2327271 |
| 1293 | ZINC15883684 | 5.4511179 |
| 1294 | ZINC26896315 | 5.5011069 |
| 1295 | ZINC92979990 | 5.3343505 |
| 1296 | ZINC72144078 | 4.6862887 |
| 1297 | ZINC25578776 | 4.7698527 |
| 1298 | ZINC16889991 | 4.556959 |
| 1299 | ZINC91270453 | 4.4227637 |
| 1300 | ZINC83230610 | 4.250337 |
| 1301 | ZINC40450041 | 4.2465881 |
| 1302 | ZINC95022749 | 4.617187 |
| 1303 | ZINC59331828 | 4.4826724 |
| 1304 | ZINC00030069 | 4.2509416 |
| 1305 | ZINC91162155 | 4.6130472 |
| 1306 | ZINC00028677 | 4.7527835 |
| 1307 | ZINC02896351 | 4.3456123 |
| 1308 | ZINC09861295 | 4.6969882 |
| 1309 | ZINC64848290 | 6.1897491 |
| 1310 | ZINC33535085 | 4.993939 |
| 1311 | ZINC09185335 | 3.947546 |
| 1312 | ZINC09861297 | 4.7354134 |
| 1313 | ZINC17197718 | 3.6867009 |
| 1314 | ZINC12411111 | 4.9049677 |
| 1315 | ZINC32826582 | 4.5725394 |
| 1316 | ZINC02278531 | 3.7456959 |
| 1317 | ZINC15828386 | 5.7350689 |
| 1318 | ZINC01064141 | 5.9041164 |
| 1319 | ZINC35448294 | 4.5826507 |
| 1320 | ZINC16969479 | 3.6229941 |
| 1321 | ZINC04739428 | 4.8853627 |
| 1322 | ZINC14359430 | 3.8280102 |
| 1323 | ZINC27546906 | 6.0526699 |
| 1324 | ZINC05126000 | 4.8038155 |
| 1325 | ZINC02887148 | 4.3599562 |
| 1326 | ZINC03051081 | 4.012849 |
| 1327 | ZINC02896526 | 3.6714579 |
| 1328 | ZINC02895957 | 5.2104692 |
| 1329 | ZINC21661071 | 4.7609104 |
| 1330 | ZINC23237284 | 4.6099004 |
| 1331 | ZINC00682495 | 5.1188676 |
| 1332 | ZINC04340036 | 4.9194786 |
| 1333 | ZINC09228813 | 4.9352196 |
| 1334 | ZINC03249012 | 4.7939875 |
| 1335 | ZINC04757793 | 4.8899214 |
| 1336 | ZINC32934569 | 5.0909619 |
| 1337 | ZINC01575402 | 4.5878547 |
| 1338 | ZINC37206688 | 4.1713002 |
| 1339 | ZINC76101685 | 5.8364578 |
| 1340 | ZINC00887484 | 5.0263924 |
| 1341 | ZINC00930555 | 4.9976247 |
| 1342 | ZINC03314268 | 5.8041882 |
| 1343 | ZINC17060393 | 5.8603019 |
| 1344 | ZINC40310117 | 5.4988172 |
| 1345 | ZINC20970164 | 5.8232066 |
| 1346 | ZINC07078310 | 3.7389864 |
| 1347 | ZINC16492771 | 3.928833 |
| 1348 | ZINC46078081 | 4.8349583 |
| 1349 | ZINC02283012 | 5.5844152 |
| 1350 | ZINC41353504 | 5.989353 |
| 1351 | ZINC21145101 | 5.1213081 |
| 1352 | ZINC01046841 | 5.1956534 |
| 1353 | ZINC12775339 | 6.5323381 |
| 1354 | ZINC04560191 | 4.9080166 |
| 1355 | ZINC01864817 | 5.4146679 |
| 1356 | ZINC66130522 | 5.4514198 |
| 1357 | ZINC45070140 | 5.3681354 |
| 1358 | ZINC21889989 | 5.1384495 |
| 1359 | ZINC02066994 | 4.6643261 |
| 1360 | ZINC09734562 | 6.0126384 |
| 1361 | ZINC00457773 | 3.9801541 |
| 1362 | ZINC74941210 | 5.6252579 |
| 1363 | ZINC91906796 | 4.0996257 |
| 1364 | ZINC13284834 | 4.3523555 |
| 1365 | ZINC67758928 | 5.0028038 |
| 1366 | ZINC95064495 | 5.0330517 |
| 1367 | ZINC37509556 | 3.6866003 |
| 1368 | ZINC72255640 | 5.0288892 |
| 1369 | ZINC89259359 | 4.7900647 |
| 1370 | ZINC92467808 | 5.2108945 |
| 1371 | ZINC03208110 | 4.2272058 |
| 1372 | ZINC35465823 | 4.1464656 |
| 1373 | ZINC92592891 | 5.3113902 |
| 1374 | ZINC71887456 | 6.8414444 |
| 1375 | ZINC28326466 | 5.157585 |
| 1376 | ZINC94097628 | 4.6194343 |
| 1377 | ZINC60349746 | 5.1194623 |
| 1378 | ZINC49472492 | 4.4556016 |
| 1379 | ZINC39929678 | 4.992918 |
| 1380 | ZINC18259878 | 4.3956515 |
| 1381 | ZINC16947985 | 4.1925686 |
| 1382 | ZINC32801532 | 4.8318392 |
| 1383 | ZINC55057221 | 4.7700881 |
| 1384 | ZINC78716799 | 6.1899797 |
| 1385 | ZINC00169015 | 4.1717239 |
| 1386 | ZINC64362184 | 5.4119171 |
| 1387 | ZINC86483064 | 4.7454471 |
| 1388 | ZINC91362117 | 4.8590318 |
| 1389 | ZINC15225414 | 3.9658891 |
| 1390 | ZINC48344572 | 4.2385344 |
| 1391 | ZINC09580096 | 4.88444 |
| 1392 | ZINC16138218 | 4.0332839 |
| 1393 | ZINC00407171 | 4.3303101 |
| 1394 | ZINC83302374 | 4.674488 |
| 1395 | ZINC28526963 | 4.9965811 |
| 1396 | ZINC72152828 | 4.891021 |
| 1397 | ZINC01080288 | 5.0549877 |
| 1398 | ZINC36369721 | 6.769576 |
| 1399 | ZINC38487402 | 5.7180315 |
| 1400 | ZINC03434395 | 6.0079658 |
| 1401 | ZINC05316100 | 5.2399214 |
| 1402 | ZINC92778997 | 4.9011394 |
| 1403 | ZINC09835648 | 6.3408567 |
| 1404 | ZINC33413799 | 3.7925243 |
| 1405 | ZINC16084837 | 5.3146393 |
| 1406 | ZINC91865351 | 4.6428281 |
| 1407 | ZINC20757713 | 6.1358869 |
| 1408 | ZINC73696260 | 5.1046721 |
| 1409 | ZINC03840167 | 6.154676 |
| 1410 | ZINC10274123 | 5.5000356 |
| 1411 | ZINC92124106 | 5.2563763 |
| 1412 | ZINC03174174 | 6.1970985 |
| 1413 | ZINC79189446 | 5.2752812 |
| 1414 | ZINC79862309 | 5.1064101 |
| 1415 | ZINC81440574 | 5.6629338 |
| 1416 | ZINC42944410 | 5.265792 |
| 1417 | ZINC40683767 | 4.7752881 |
